# Metabolomics and polysaccharide fingerprinting reveal compositional differences between cultivated Fritillaria bulbs and flower by-products

**DOI:** 10.1016/j.fochx.2026.104348

**Published:** 2026-08-21

**Authors:** Zhao Geng, Yijie Ren, Zhen Hu, Chengjun He, Donglin Gu, Zhichun Zhang, Yan Gou, Jingzhe Pu, Yazhong Zhang, Lei Yang, Jincai Lu

**Affiliations:** a Sichuan Institute for Drug Control (Sichuan Testing Center of Medical Devices, Sichuan Institute of Musk Deer Breeding), Chengdu 611731, China; b Anhui Institute for Food and Drug Control (NMPA Key Laboratory for Quality Research and Evaluation of Traditional Chinese Medicine), Hefei 230051, China; c Sichuan Key Laboratory of Fruit and Vegetable Postharvest Physiology, College of Food Science, Sichuan Agricultural University, Ya'an 625014, China; d School of Traditional Chinese Materia Medica, Shenyang Pharmaceutical University, Shenyang 110016, China

**Keywords:** Cultivated Fritillariae Cirrhosae Bulbus, Flower by-products, UHPLC-Q-TOF-MS metabolomics, Alkaloid quantification, Polysaccharide characterization, Compositional profiling

## Abstract

Although Fritillariae Cirrhosae Bulbus (FCB) is used in food-related product contexts and local botanical-infusion practices, co-produced flowers remain underutilized and poorly characterized. To support QC-oriented screening and consistency evaluation, we compared bulbs and flowers from two dominant cultivated sources, *F. unibracteata* var. *wabuensis* (FW) and *F. unibracteata* (F), across 36 batches. UHPLC-Q-TOF-MS metabolomics annotated 76 compounds and discriminated between cultivated source and tissue, yielding candidate markers. Targeted UHPLC-QQQ-MS quantified five alkaloids; peiminine differed significantly across four sample groups, whereas sipeimine showed a higher distributional tendency in FW flowers, indicating that flower by-products should be evaluated as compositionally distinct materials rather than simple bulb substitutes. GPC-RI-MALS indicated higher mean PDI in flower crude polysaccharides, and a validated UHPLC-QQQ-MS method quantified 12 monosaccharides, of which eight were higher in flowers. This framework provides integrated chemical evidence for FW/F discrimination and a compositional basis for future QC-oriented and food-related evaluation of flower by-products.

## Graphical abstract

**Figure:**
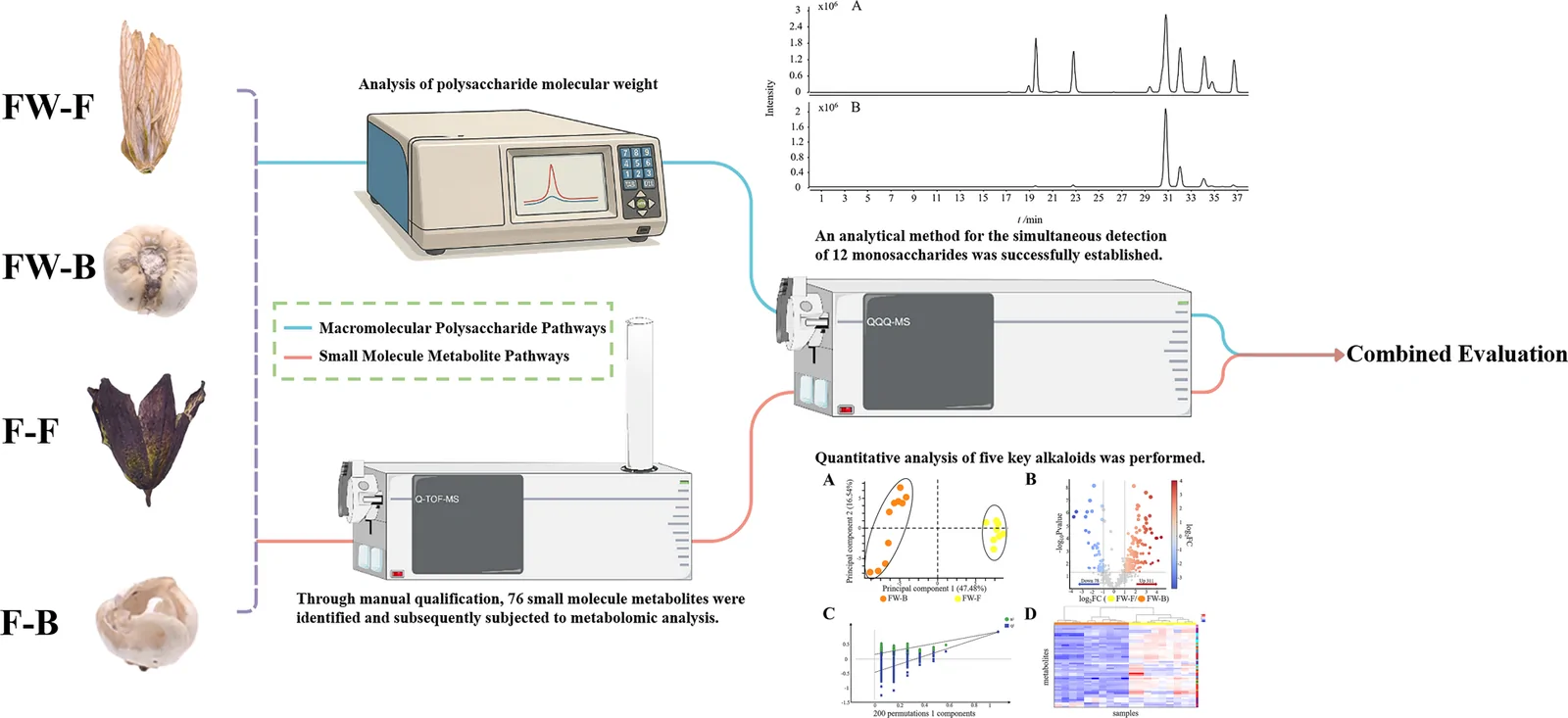

## 1 Introduction

Fritillariae Cirrhosae Bulbus (FCB) is primarily known as a traditional botanical material and has also been reported in food-related products and botanical-infusion contexts. It is primarily produced in the high-altitude regions of western Sichuan and northwestern Yunnan (Lu et al., 2022). Rising demand and limited wild supply-due to slow natural regeneration and long-term overharvesting (Li et al., 2017; Lu et al., 2022)-have accelerated the transition to cultivated production. Under mounting pressure on wild resources, cultivation has become a key source of supply (Ma et al., 2021). As a result of this supply-demand imbalance, FCB has also been reported to command a marked price premium, being priced tens of times higher than other commonly traded Fritillaria bulbs (e.g., *Fritillaria thunbergii* Miq. (FTB) and *Fritillaria ussuriensis* Maxim. (FUM)), thereby creating economic incentives for market substitution and adulteration. This market pressure underscores the need for fit-for-purpose compositional screening and consistency evaluation in relevant product formats. This issue is particularly pronounced in processed products (e.g., powders), where diagnostic morphology is largely lost, and composition and batch-to-batch consistency become difficult to assess by visual inspection alone (Du et al., 2021). Current standards and commercial practice recognize six botanical sources of FCB. Among them, *Fritillaria unibracteata* Hsiao et K.C.Hsia var. *wabuensis* (FW; bulb: FW-B; flower: FW-F) and *Fritillaria unibracteata* Hsiao et K.C.Hsia (F; bulb: F-B; flower: F-F) are the two closely related and dominant cultivated sources, accounting for the largest planted area and highest yields (Wang et al., 2020). However, their high degree of similarity blurs the distinction between them in commercial trade, particularly in processed products, thereby increasing the need for evidence-based chemical discrimination and fit-for-purpose quality evaluation (Gui et al., 2023; Ji et al., 2024). Historically, FW was at times treated as a variant of F, contributing to practical ambiguity in trade.

Despite the clear practical relevance of distinguishing these two cultivated sources, systematic food-relevant chemical comparisons and consistency-oriented evaluation strategies remain scarce. In the absence of an evidence-based quality evaluation toolkit, it is difficult to precisely differentiate them (Jiang, Li, et al., 2024). In practice, FW and F may also be traded under different commercial grades, further increasing the need for evidence-based discrimination in quality control. Accurate discrimination is essential not only to deter economically motivated substitution or adulteration, but also to support fit-for-purpose quality control, raw-material consistency management, and product standardization when materials are processed, and morphology is not informative, and to provide a compositional basis for future food-related evaluation when key constituents (e.g., steroidal alkaloids) vary across sources and organs (bulbs vs. flowers).

Most previous studies have centered on small-molecule compounds, particularly steroidal alkaloids. However, the reported total alkaloid content in *Fritillaria* bulbs is remarkably low (approximately 0.19-0.25%, *w*/w) (Guo et al., 2025; Ma et al., 2021). By comparison, polysaccharides are present at substantially higher levels, with reported contents reaching up to 7.01% (*w*/w) (Jiang, Wang, et al., 2024), and may therefore contribute additional macromolecular information for compositional differentiation across cultivated sources and tissues. Nevertheless, current quality control criteria often rely on a single alkaloid index, resulting in the underrepresentation of macromolecular carbohydrates in routine evaluation (Borjigin et al., 2023; Ji et al., 2024).

Even within polysaccharides research, monosaccharide profiling has often been restricted to a limited panel of 5-8 common sugars (Abuduwaili et al., 2019; Pan et al., 2018; Rozi et al., 2019; Zhang et al., 2020), potentially obscuring the compositional heterogeneity of the complex polysaccharide system. In contrast, the present workflow provides a more information-rich compositional fingerprint for complex polysaccharides and thus offers stronger analytical support for their compositional characterization, quality control, and source differentiation. Although FCB is typically marketed as the bulb, cultivation of its botanical sources generates substantial aerial biomass, which is typically discarded and remains insufficiently characterized; a key question for future food-related evaluation is whether flowers differ chemically from bulbs in compositionally meaningful ways (e.g., alkaloid burden and polysaccharide heterogeneity). Reports from cultivation bases suggest that dried flowers may be steeped locally as botanical infusions, whereas systematic compositional characterization of the flower material remains limited. In addition, studies on the flowers of FTB (Li et al., 2022), FUM (Xu et al., 1988), and *Fritillaria hupehensis* Hsiao et K. C. Hsia (Pi et al., 2007) have suggested that the floral parts may contain steroidal alkaloids also reported in bulbs, suggesting that flower material warrants systematic compositional characterization and further food-related evaluation before broader utilization.

These considerations motivated an integrated strategy that couples small-molecule metabolomics with refined polysaccharide characterization. Leveraging the high resolution of Ultra-High Performance Liquid Chromatography Quadrupole Time-of-Flight Mass Spectrometry (UHPLC-Q-TOF-MS), we conducted systematic qualitative profiling of small-molecule compounds (Ren et al., 2024) and applied an untargeted metabolomics workflow to compare metabolic differences and identify candidate markers for ingredient-identity screening (Jiang et al., 2024, Liu et al., 2020). To support the interpretation of representative alkaloid-related differences, we performed targeted UHPLC-QQQ-MS quantification of five representative steroidal alkaloids. For polysaccharides, we optimized the PMP-derivatization scheme and detection parameters (Yang et al., 2016), thereby establishing and validating a UHPLC-QQQ-MS method capable of simultaneous quantification of 12 monosaccharides. In parallel, gel permeation chromatography coupled with refractive index and multi-angle light scattering detection (GPC-RI-MALS) was employed to characterize molecular-weight distributions (Cheong et al., 2015; Zhao et al., 2018).

Accordingly, this work was designed as a QC-oriented compositional profiling study to delineate the chemical divergence between two taxonomically authenticated dominant cultivated sources (FW vs. F) and between tissues (bulbs vs. flowers), thereby establishing a compositional baseline for future evaluation of flower by-products. The novelty of this study lies in the integration of positive-ion-responsive untargeted metabolomics, targeted UHPLC-QQQ-MS quantification of representative steroidal alkaloids, expanded 12-monosaccharide profiling, and GPC-RI-MALS-based crude-polysaccharide molecular-weight distribution analysis within the same 36-batch sample set. This integrated workflow enables both source-level and tissue-level comparison and provides a preliminary candidate compositional marker panel for QC-oriented screening and future targeted validation.

## 2 Experimental

### 2.1 Sample collection

In this study, F and FW materials, including bulbs and their corresponding co-produced flowers, were collected from five cultivation bases in April 2025. Plant materials were taxonomically authenticated by Jingliang Qi from the Sichuan Institute for Drug Control. Sample IDs, sample types, and cultivation bases are listed in **Table S1**. After collection, all samples were dried at 40 °C to constant weight and stored at -20 °C until analysis. Each batch represented a separately collected and coded plant-material batch from the corresponding cultivation source rather than an analytical duplicate. A total of 36 batches were collected, including FW-B (*n* = 10), FW-F (*n* = 9), F-B (*n* = 10), and F-F (*n* = 7) (**Table S1**; representative photographs are shown in Fig. 1). The number of batches contributed by each cultivation base is provided in **Table S1**. All samples were collected within the same collection period to minimize seasonal variation.

**Fig. 1:**
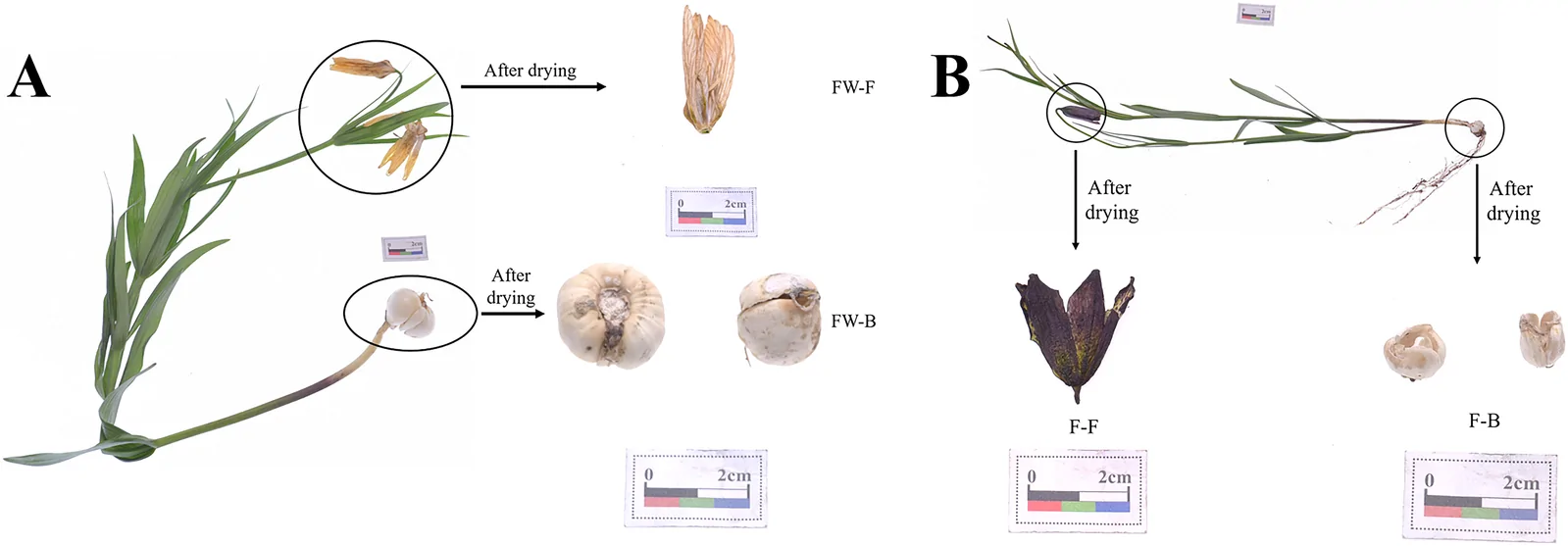
Representative photographs of cultivated Fritillaria materials and the corresponding dried samples. (A) *Fritillaria unibracteata* Hsiao et K.C.Hsia var. *wabuensis* (FW; bulb: FW-B; flower: FW-F) and (B) *Fritillaria unibracteata* Hsiao et K.C.Hsia (F; bulb: F-B; flower: F-F).

### 2.2 Chemicals and reagents

Throughout all experiments, ultrapure water was obtained from a Milli-Q ultrapure water system. For GPC-RI-MALS, the mobile phase was prepared using commercially available distilled water (Watsons). HPLC-grade acetonitrile, methanol, ammonium acetate, ammonium formate, trifluoroacetic acid (TFA), and formic acid were purchased from Fisher Scientific. Analytical-grade ammonia solution, acetic acid, diethyl ether, and chloroform were purchased from Chron Chemicals. 1-Phenyl-3-methyl-5-pyrazolone (PMP) was purchased from Shanghai Yuanye Bio-Technology Co., Ltd. The 18 reference standards used for UHPLC-Q-TOF-MS qualitative analysis, the dextran standards used for GPC-RI-MALS, the five reference standards used for UHPLC-QQQ-MS alkaloid quantification, and the 12 monosaccharide standards are listed in **Table S2**.

### 2.3 UHPLC-Q-TOF-MS analysis

#### 2.3.1 Sample preparation

For untargeted metabolomics, all 36 batches of samples were prepared and analyzed by UHPLC-Q-TOF-MS. Exactly 1.0 g of pulverized sample was weighed, and 25 mL of 80% (*v*/v) methanol was added by weight. The mixture was then sonicated for 30 min. The extract was allowed to cool to room temperature and reweighed. Any weight loss was made up with 80% methanol, and the mixture was shaken well and filtered (0.22 μm).

Reference standards: Individual stock solutions were prepared by accurately weighing each reference standard, dissolving it in 80% methanol, and making up to a final volume of 10 mL. Stock solutions were stored under refrigerated conditions until use. Prior to analysis, a mixed reference-standard working solution was freshly prepared by combining the individual stocks to give an approximate concentration of 10 μg/mL for each analyte.

#### 2.3.2 Optimization of UHPLC-Q-TOF-MS conditions

Before chemical profiling, UHPLC-Q-TOF-MS conditions were optimized to maximize signal response and chromatographic resolution. Three aqueous phases and two organic phases were tested, together with two candidate columns, and separation performance was compared. Extraction solvent optimization was also performed by comparing 30% methanol, 50% methanol, pure methanol, 80% ethanol, and 80% methanol. Among these solvents, 80% methanol was selected because it provided stronger MS response and more detectable chromatographic peaks under the optimized UHPLC-Q-TOF-MS conditions (**Fig. S1**). The optimal configuration used 0.1% formic acid in water and methanol as the mobile phases on a Thermo Hypersil GOLD aQ column (2.1 × 150 mm, 1.9 μm), which provided both stronger MS responses and improved peak separation. The final high-organic segment of the gradient was used to elute late-retained constituents.

#### 2.3.3 Conditions for qualitative analysis

The chromatography was performed on an Agilent 1290 UHPLC system coupled to an Agilent 6540 Q-TOF mass spectrometer (Agilent Co., Santa Clara, CA, USA). Data were acquired using MassHunter Workstation Software (LC/MS Data Acquisition Version B.08.00). Qualitative data analysis was performed using the MassHunter Workstation Software (Qualitative Analysis Version B.07.00). Analytes were separated on a Thermo Hypersil GOLD aQ column (2.1 mm × 150 mm, 1.9 μm). The column temperature was kept at 35 °C. The mobile phase in this study consisted of 0.1% formic acid in water (eluent A) and methanol (eluent B). The injection volume was 1.0 μL, and the injected sample solution corresponded to 40 mg starting material/mL before membrane filtration. The flow rate was 0.3 mL/min. The gradient elution was performed as follows: 0 min–15.5 min, 95%–50% A; 15.5 min–25 min, 50%–45% A; 25 min–42 min, 45%–5% A; 42 min–47 min, 5% A. Electrospray ionization (ESI) was used for mass spectrometry detection in positive and negative-ion modes. The mass spectrometer parameters were set as follows: Gas Temp: 325 °C; Gas Flow: 8 L/min; Nebulizer: 35 psig; Sheath Gas Temp: 350 °C; Sheath Gas Flow: 11 L/min; Fragmentor: 110 V; VCap: Pos 4000 V / neg 3500 V; Nozzle Voltage: Pos 0 V / neg 1000 V; MS scan range: *m/z* 100–1600. MS/MS spectra were acquired in data-dependent auto-MS/MS mode, and collision energies (CEs) were set to 10, 20, and 40 V.

#### 2.3.4 Untargeted metabolomics analysis

Because positive-ion mode showed stronger overall ion responses and more abundant discernible chromatographic signals than negative-ion mode under the present UHPLC-Q-TOF-MS conditions, positive-ion-mode data were used for downstream metabolomic analysis (**Figs. S2 and S3**). This mode provided more informative responses for major Fritillaria-related constituents, especially steroidal alkaloid-related features. Therefore, the downstream metabolomic comparisons should be interpreted as mainly covering positive-ion-responsive features rather than the complete metabolome.

Multivariate analyses (principal component analysis (PCA) and hierarchical cluster analysis (HCA)) were performed in MS-DIAL using the aligned peak-area matrix. OPLS-DA in MS-DIAL was used to obtain VIP scores for feature interpretation, whereas model reporting and validation were performed in SIMCA-P using the exported aligned peak-area matrix, including R^2^X(cum), R^2^Y(cum), Q^2^(cum), CV-ANOVA *p* values, and permutation testing (*n* = 200). In addition, univariate statistics were conducted based on fold change (FC) and a two-sided unpaired Student's *t*-test. Volcano plots were generated with custom Python scripts using log_2_FC and -log10(p) for visualization; features meeting |log_2_FC| ≥ 1 and *p* < 0.05 were used for exploratory feature screening. Candidate features were subsequently prioritized by integrating OPLS-DA VIP scores, fold change, peak quality, MS/MS interpretability, curated-library matching, and manual annotation confidence. Candidate compounds were further curated by excluding redundant isotope/adduct signals, poorly resolved peaks, and entries lacking clear, coherent annotation-supporting MS/MS evidence. An in-house curated chemical library of FCB-related compounds was constructed prior to data mining. Eighteen authentic reference standards (**Table S2**) were analyzed under the same UHPLC-Q-TOF-MS conditions, and compounds with available standards were confirmed by matching retention time (RT), accurate precursor *m/z*, and MS/MS product-ion spectra. Based on these confirmed standards, diagnostic product ions and characteristic neutral losses were summarized to establish class-specific fragmentation rules, which, together with literature-reported fragmentation pathways/spectra, were used to putatively annotate additional FCB-related constituents in the curated library. Differential features prioritized by jointly considering volcano-based screening and OPLS-DA VIP scores (VIP > 1) were matched against the curated library using accurate mass and MS/MS fragmentation, and further confirmation (RT and/or MS/MS matching) was performed when the corresponding reference standards were available. Metabolite annotation confidence was assigned as Level 1 when compounds were confirmed by authentic standards through matching RT, accurate precursor *m/z*, and MS/MS spectra, and as Level 2 when compounds were putatively annotated based on accurate mass and literature-consistent MS/MS fragmentation evidence.

#### 2.3.5 Stability study of the UHPLC-Q-TOF-MS system

In the data acquisition process, the stability of the instrumental system was crucial for obtaining precise retention times and *m/z* values of the compounds, which directly affected the outcomes of subsequent multivariate statistical analyses. To evaluate this stability, Quality Control (QC) samples were periodically analyzed.

A pooled QC sample was prepared by mixing aliquots of all study samples and was injected once before the sequence and then after every nine study-sample injections (five QC injections in total). The total ion chromatogram (TIC) acquisition time was approximately 47 min. For stability assessment, the chromatogram was divided into five segments (approximately 9–10 min each). Within each window, representative peaks were integrated using identical extracted-ion chromatogram (EIC) settings. Twenty ion peaks were extracted in total, and RSDs of peak areas and retention times were calculated. The analytical sequence of the study samples was randomized.

The predefined acceptance criteria were RT RSD < 0.5%, *m/z* RSD ≤ 0.05‰, and EIC peak-area RSD < 15% across QC injections. System stability metrics met these criteria and are summarized in **Table S3**.

### 2.4 UHPLC-QQQ-MS analysis

The chromatography was performed on an Agilent 1290 UHPLC system coupled to an Agilent 6460 QQQ mass spectrometer (Agilent Co., Santa Clara, CA, USA). Data were acquired using Agilent MassHunter (Ver. B.08.00) and processed using MassHunter Workstation (Ver. B.07.01).

#### 2.4.1 Sample preparation for alkaloid quantification

0.1 g of powdered sample was accurately weighed into a stoppered conical flask, and 1 mL of concentrated ammonia solution was added; the mixture was allowed to stand for 1 h. A mixed solvent of chloroform-diethyl ether-ethanol (25:8:2.5, *v*/v/v; 40 mL) was added, and the mixture was refluxed for 2 h. After cooling to room temperature, the extract was filtered, and the filtrate was transferred to a 50 mL volumetric flask. The residue was rinsed 2–3 times with the same solvent, and the combined filtrates were made up to volume and mixed. An aliquot (5 mL) was evaporated to dryness in a water bath, reconstituted in methanol, brought to 5 mL, and filtered through a 0.22 μm membrane for analysis (Geng et al., 2018).

Reference standards: Individual stock solutions were prepared by accurately weighing each reference standard, dissolving it in methanol, and making up to a final volume of 10 mL. Stock solutions were stored at 4 °C until analysis.

#### 2.4.2 Conditions for alkaloid quantification

Analytes were separated on an Agilent Eclipse XDB C18 (2.1 mm × 100 mm, 1.8 μm). The column temperature was kept at 35 °C. The mobile phase in this study consisted of 10 mmol/L ammonium formate solution containing 0.1% formic acid (eluent A) and acetonitrile (eluent B). The injection volume was 1.0 μL. The flow rate was 0.4 mL/min. The gradient elution was performed as follows: 0 min–2 min, 10%–20% B; 2 min–11 min, 20% B. ESI was used for mass spectrometry detection in positive-ion mode. The mass spectrometer parameters were set as follows: Gas Temp: 325 °C; Gas Flow: 9 L/min; Nebulizer: 40 psig; Sheath Gas Temp: 350 °C; Sheath Gas Flow: 10 L/min; Cap: 4000 V; Nozzle Voltage: 0 V.

#### 2.4.3 Sample preparation for monosaccharide composition analysis

Sample preparation for monosaccharide composition analysis consisted of three parts. Part 1 (polysaccharides extraction): 1 g of sample was accurately weighed, 20 mL of 80% ethanol was added, and the mixture was refluxed at 87 °C for 1 h. After filtration, the residue was washed three times with 10 mL hot 80% ethanol and air-dried. Ultrapure water (50 mL) was then added, and the mixture was refluxed at 100 °C for 1 h. The filtrate was collected, and four volumes of absolute ethanol were added dropwise under stirring. The mixture was precipitated at 4 °C for 12 h, centrifuged, and the precipitate was re-dissolved in 10 mL ultrapure water to obtain a polysaccharide solution. Part 2 (hydrolysis): 250 μL polysaccharide solution was mixed with an equal volume of 4 mol/L TFA and hydrolyzed at 110 °C for 4 h, then evaporated to dryness under nitrogen. Part 3 (PMP derivatization): the residue was reconstituted with 250 μL ultrapure water, mixed with an equal volume of 0.5 mol/L PMP in methanol, and derivatized at 70 °C for 1.5 h. After derivatization, 250 μL glacial acetic acid was added, and the mixture was evaporated to dryness under nitrogen. The residue was then extracted three times with chloroform after the addition of 1.5 mL ultrapure water. Prior to injection, samples were filtered through a 0.22 μm membrane.

Reference standards: Individual stock solutions were prepared by accurately weighing each reference standard, dissolving it in water, and making up to a final volume of 10 mL. Stock solutions were stored at 4 °C until analysis. Reference standards were subjected to the same PMP-derivatization procedure as samples prior to UHPLC-QQQ-MS analysis. The quantified monosaccharide contents in samples were normalized to the starting sample weight and expressed as mg/g starting material for reporting.

#### 2.4.4 Conditions for monosaccharide quantification

Analytes were separated on an ACQUITY UPLC® BEH C18 (2.1 mm × 150 mm, 1.7 μm). The mobile phase in this study consisted of 5 mmol/L ammonium acetate solution containing 0.1% formic acid (eluent A) and acetonitrile (eluent B). The injection volume was 1.0 μL. The flow rate was 0.3 mL/min. The gradient elution was performed as follows: 0 min–22 min, 89%–80% A; 22 min–35 min, 80% A; 35 min–35.1 min, 80%–10% A; 35.1 min–38 min, 10% A. ESI was used for mass spectrometry detection in positive-ion mode. The mass spectrometer parameters were set as follows: Gas Temp: 330 °C; Gas Flow: 11 L/min. Other parameters were identical to those used for alkaloid quantification. In addition, the parameters of multiple reaction monitoring (MRM) were optimized (**Table S4**).

For quantitative profiling of polysaccharide-derived monosaccharides, method optimization was also carried out on the UHPLC-QQQ-MS platform, with particular attention to the chromatographic separation of monosaccharide isomers. By jointly optimizing the mobile phase system, column selection, and PMP derivatization conditions, simultaneous quantification of 12 monosaccharides was achieved (**Fig. S4-S7**).

#### 2.4.5 Method validation and current-batch confirmation of UHPLC-QQQ-MS

For the monosaccharide assay, the UHPLC-QQQ-MS method was validated by evaluating linearity, limits of detection (LODs), limits of quantification (LOQs), precision, stability, repeatability, and recovery.

For monosaccharide quantification, calibration curves were established by analyzing a series of mixed reference standard solutions with various desired concentrations. The LODs and LOQs were determined by analyzing serially diluted mixed reference standard solutions of each compound until the signal-to-noise (S/N) ratios reached approximately 3 and 10, respectively. The same mixed reference standard solution was tested six times to assess precision. Repeatability was evaluated with six independently prepared samples. Stability was assessed by analyzing the same mixed reference standard solution and sample solution at different time points (0, 2, 4, 6, 8, 10, and 12 h). The RSD values of the corresponding peak areas were calculated to evaluate the results of the aforementioned tests. Accuracy evaluation was conducted through a recovery experiment. Although stable isotope-labelled monosaccharide internal standards or matrix-matched calibration curves were not used, both reference standards and sample hydrolysates underwent the same PMP-derivatization procedure before UHPLC-QQQ-MS analysis. The monosaccharide assay was validated in representative flower and bulb matrices in terms of linearity, precision, solution stability, repeatability, recovery, LOD, and LOQ **(Table S5)**, supporting reliable comparative quantification across the sample set. An individual reference standard with a known amount was added to a sample. The recovery value (%) was calculated using the following equation: recovery (%) = (detected amount - original amount) / spiked amount × 100%.

For alkaloid quantification, the UHPLC-QQQ-MS procedure was adopted from a previously validated method and was applied under the same instrumental platform, chromatographic column, and chromatographic and mass-spectrometric conditions. Therefore, the alkaloid assay was not re-established as a new quantitative method in the present study. To confirm its suitability for the current analytical batch, the linear range and instrumental precision were re-checked, and the results are summarized in **Table S6.** Validation performance for the monosaccharide assay, including linearity, precision, stability, repeatability, recovery, and sensitivity, is summarized in **Table S5,** and all validation parameters met acceptance criteria. Current-batch applicability confirmation results for the alkaloid assay, including linearity and instrumental precision, are summarized in **Table S6**, and both confirmation parameters met acceptance criteria.

### 2.5 GPC-RI-MALS analysis

#### 2.5.1 Sample preparation

Non-hydrolyzed polysaccharide samples were used for GPC analysis; samples were prepared according to Part 1 in Section 2.4.3 and analyzed by GPC-RI-MALS.

#### 2.5.2 Conditions

An Agilent 1200 HPLC system that was equipped with a Wyatt Optilab T-rEX refractive index (RI) detector, a Wyatt DAWN HELEOS II multi-angle light scattering (MALS) detector (Wyatt Technology, Santa Barbara, CA, USA), and an Agilent diode-array detector (DAD; Agilent Technologies, USA) was used. Two gel-permeation columns (Shodex SB-804 HQ, 300 mm × 8.0 mm, 10 μm; and Shodex SB-806 HQ, 300 mm × 8.0 mm, 13 μm) were connected in series. The mobile phase was 0.1 M NaCl, with the column maintained at 40 °C. The flow rate was 0.5 mL/min, and the injection volume was 100 μL.

The MALS detector was fitted with a quartz flow cell, and operated at a laser wavelength of 658 nm. The 90° detector was calibrated with toluene, and the remaining angle detectors were normalized using dextran D5 (Mp = 36,800 g/mol). The refractive index increment (dn/dc) was set to 0.138 for all samples to support comparative molecular-weight distribution analysis under identical calculation conditions. The RI calibration constant was obtained using a series of NaCl standard solutions. The DAD wavelength was set to 280 nm as an auxiliary monitoring channel for potential proteinaceous co-eluting components that could affect RI/MALS-based molecular-weight interpretation.

### 2.6 Statistical analysis

Each independently collected batch was treated as an independent batch-level replicate for statistical analysis, with group sizes of FW-B (*n* = 10), FW-F (*n* = 9), F-B (*n* = 10), and F-F (*n* = 7). Unless otherwise stated, statistical analyses were performed at the batch level. For targeted alkaloid and monosaccharide quantification, overall differences among the four sample groups were evaluated using the Kruskal-Wallis test. Pairwise comparisons were performed using Dunn's post hoc test, and *p* values were adjusted using the Benjamini-Hochberg method to obtain q values. Epsilon-squared (ε^2^) was calculated as an effect-size estimate for the Kruskal-Wallis test. To explore cross-layer relationships, Spearman rank correlation analysis was performed across the 36 matched batches between targeted alkaloids, GPC-RI-MALS-derived weight-average molecular weight (*M*_w_) and polydispersity index (PDI) values, and quantifiable polysaccharide-derived monosaccharides. Sorbose was excluded because it was not detected across the sample set. Correlation *p* values were adjusted using the Benjamini-Hochberg method, and the results were visualized as a correlation heatmap.

## 3 Results and discussion

### 3.1 Chemical profiling of the four sample groups

The TICs acquired in both positive and negative-ion modes for the four sample groups indicated that the positive-ion mode yielded a richer signal profile with well-defined peak shapes (**Fig. S2**). Therefore, the positive-ion data were used for subsequent profiling. Based on information from reference standards and the literature, we systematically summarized the fragmentation patterns of major chemical classes. This provided additional MS/MS evidence to complement database matching and supported the construction of a curated chemical library of FCB-related compounds for subsequent metabolomics analysis (Theodoridis et al., 2023). In total, 76 compounds were annotated across the four sample groups (FW-B, FW-F, F-B, and F-F) by matching MS/MS evidence to the curated chemical library of FCB-related compounds. Notably, 24 alkaloids were detected in FW-F and F-F samples, including sipeimine-3β-D-glucoside, peimine, peiminine, and sipeimine, which are widely regarded as characteristic constituents and routinely monitored quality markers of FCB. In addition, multiple non-alkaloid metabolites were annotated or putatively annotated according to the evidence levels listed in **Table S7**, including 13 flavonoids, 9 amino acids, 10 organic acids, 5 nucleosides, 2 vitamins, and 3 phenylpropanoids, together with minor amounts of saponins, coumarins, nucleobases, sucrose esters, terpenoids, and lignans (**Table S7**). Compounds confirmed using authentic standards by matching RT, accurate precursor *m/z*, and MS/MS spectra were assigned as Level 1 annotations, whereas compounds annotated based on accurate mass and literature-consistent MS/MS fragmentation evidence were assigned as Level 2 putative annotations (**Table S7**). Therefore, Level 2 compounds were used primarily to describe chemical-class coverage and to generate candidate markers, rather than as fully confirmed compound identities.

#### 3.1.1 MS/MS-based annotation of representative compounds

##### 3.1.1.1 Fragmentation patterns of alkaloids

Alkaloids in *Fritillaria*, such as peimine and sipeimine-3β-D-glucoside, are widely regarded as characteristic marker constituents and are routinely monitored as quality markers (Zhuang et al., 2025). Here, sipeimine-3β-D-glucoside was used not as a newly identified differential compound but as a standard-confirmed representative alkaloid to establish diagnostic MS/MS fragmentation rules for subsequent annotation; differential candidate compounds are summarized in Section 3.2.2 and **Tables S8–S11**.

In positive-ion mode, compound 27 exhibited a quasi-molecular ion at *m/z* 592.3876 [M + H]^+^. Major product ions included *m/z* 574.3802, 412.3226, 138.1284, and 112.1126. The fragment at *m/z* 574.3802 was assigned to the loss of one H_2_O molecule from the precursor ion, and the ion at *m/z* 412.3226 was assigned to the sequential loss of H_2_O and C_6_H_10_O_5_, yielding a deglycosylated fragment. The ions at *m/z* 138.1284 and 112.1126 were tentatively attributed to the neutral losses of C_26_H_41_O_6_ and C_27_H_43_O_7_, respectively. Based on these fragmentation features and confirmation with an authentic reference standard, compound 27 was identified as sipeimine-3β-D-glucoside (C_33_H_53_NO_8_) (Fig. 2A).

**Fig. 2:**
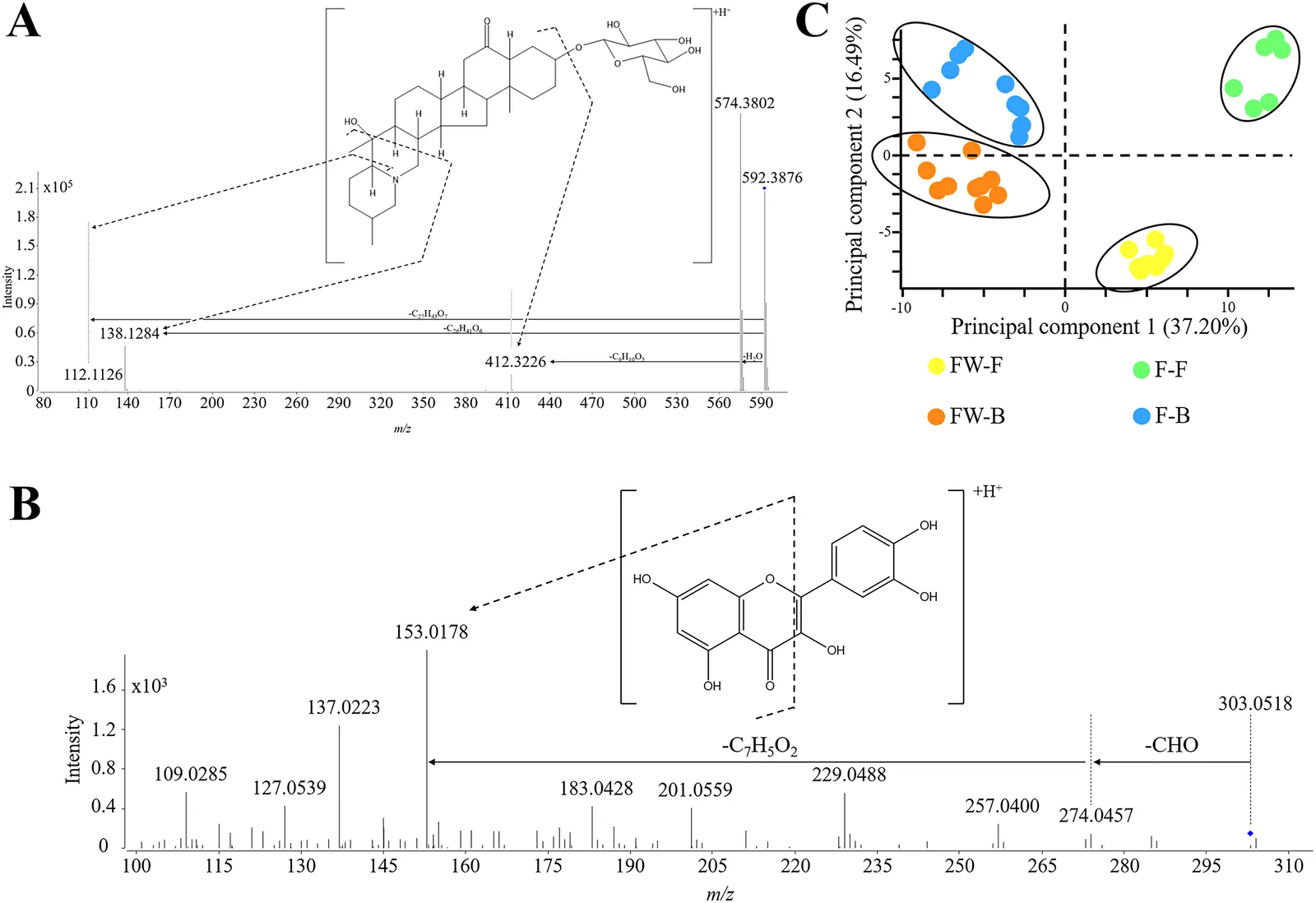
Representative MS/MS spectra for compound annotation and global PCA score plot of untargeted metabolomic profiles. (A) MS/MS spectrum and structural assignment of sipeimine-3β-D-glucoside (compound **27**) in positive-ion mode. (B) MS/MS spectrum and structural assignment of quercetin (compound **57**) in positive-ion mode. (C) PCA score plot across four sample groups (FW-B, FW-F, F-B, and F-F).

##### 3.1.1.2 Fragmentation patterns of flavonoids

Flavonoids frequently occur as glycosides or free aglycones, and their MS/MS spectra often feature characteristic neutral losses of sugar moieties and retro-Diels-Alder (RDA) cleavages. Here, quercetin is used as a representative example to illustrate the fragmentation behavior of flavonoid compounds.

In positive-ion mode, compound **57** displayed a quasi-molecular ion at *m/z* 303.0518 [M + H]^+^, with major product ions at *m/z* 274.0457 and 153.0178. The ion at *m/z* 274.0457 was generated by loss of a CHO radical from the precursor, and subsequent RDA cleavage produced *m/z* 153.0178 via loss of C_7_H_5_O_2_. Together with an authentic standard and the literature (Ke et al., 2016), compound **57** was identified as quercetin (C_15_H_10_O_7_) (Fig. 2B).

Fragmentation characteristics of representative organic acids, coumarins, nucleosides, and amino acids are summarized in **Fig. S8–S11**.

### 3.2 Untargeted metabolomics analysis based on UHPLC-Q-TOF-MS

With the curated chemical library established, untargeted metabolomics was performed to compare small-molecule profiles across cultivated sources (FW vs. F) and tissues (bulb vs. flower), and to screen for potential discriminant markers. Because the positive-ion mode produced more features with better peak shapes, subsequent statistical analyses were mainly based on the positive-mode data. PCA was first used to examine overall separation among the four groups (FW-B, FW-F, F-B, and F-F) based on UHPLC-Q-TOF-MS data. OPLS-DA was then applied to identify variables contributing most to group discrimination. In addition, volcano plots and hierarchical clustering analysis (HCA) were performed at the feature level (*m/z*-RT features). High-confidence differential compounds obtained after manual curation and matching against the curated chemical library are summarized in **Tables S8-S11**.

#### 3.2.1 Multivariate statistics reveal metabolomic clustering patterns among Fritillaria sample groups

First, unsupervised PCA was performed to assess global metabolomic variation and within-group clustering. The PCA score plot showed discernible separation trends among groups in the principal component space **(**Fig. 2C). The first two principal components explained 53.69% of the total variance, indicating that PCA captured the major separation trends while additional variance remained distributed across higher-order components. This residual variance may reflect cultivation-source/batch heterogeneity and lower-abundance positive-ion-responsive features. Therefore, PCA was used as an exploratory overview rather than as the sole basis for marker selection.

To separate tissue effects (bulb vs. flower) from source effects (FW vs. F), four pairwise comparisons were defined for downstream analyses: (1) FW-B vs. FW-F, (2) F-B vs. F-F, (3) FW-B vs. F-B, and (4) FW-F vs. F-F.

For each comparison, PCA and OPLS-DA models were established to enhance discriminative power and to identify variables contributing most to group separation. All four PCA models showed separation trends (Fig. 3, Fig. 4, Fig. 5, Fig. 6). The OPLS-DA models were evaluated using R^2^X(cum), R^2^Y(cum), Q^2^(cum), CV-ANOVA *p* values, and permutation tests (*n* = 200) to assess model validity and potential overfitting (Fig. 3, Fig. 4, Fig. 5, Fig. 6). The corresponding model parameters, Q^2^ intercepts, and CV-ANOVA *p* values are provided in the figure notes. Among the four pairwise comparisons, the bulb-to-bulb comparison between FW-B and F-B showed comparatively weaker model validation performance, with Q^2^(cum) only slightly above 0.5 and a non-significant CV-ANOVA result (*p* = 0.150). Because no external validation cohort was available in the present study, the features obtained from this comparison were interpreted cautiously as preliminary exploratory candidates requiring independent validation rather than robust discriminant markers. Building on multivariate screening, univariate statistics were applied to refine differential features. Fold change and two-sided Student's *t*-test were used to assess effect size and significance, and volcano plots were generated using |log_2_FC| ≥ 1 and -log10(p) for visualization, while significance thresholds were defined as |log_2_FC| ≥ 1 and *p* < 0.05 (Fig. 3, Fig. 4, Fig. 5, Fig. 6). HCA results (Fig. 3, Fig. 4, Fig. 5, Fig. 6) provided a complementary visualization of sample-to-sample similarity, with clearer groupwise clustering in tissue-related comparisons and comparatively weaker separation in the FW-B versus F-B comparison.

**Fig. 3:**
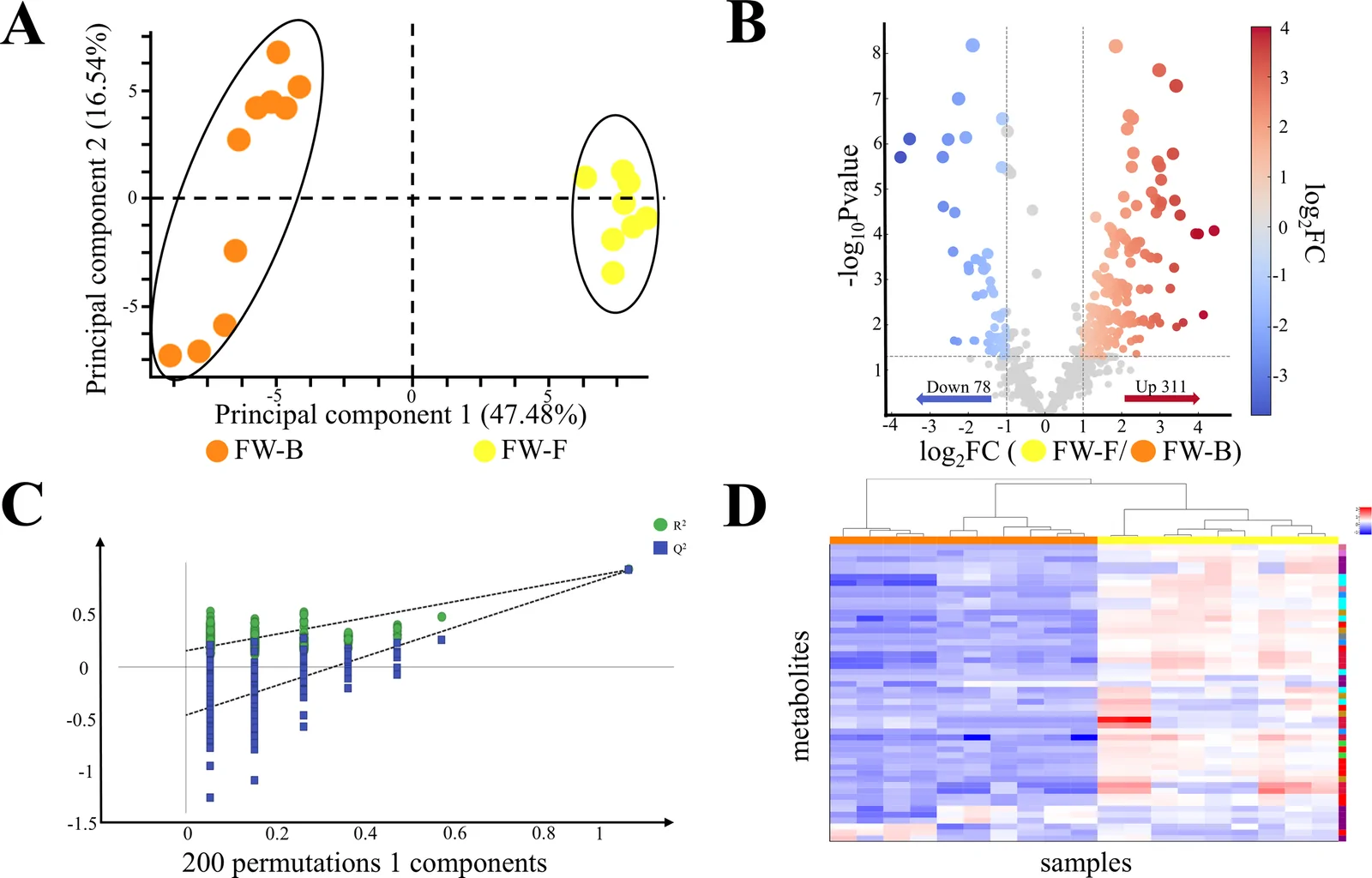
Metabolomic differences between bulbs and flowers of cultivated FW (FW-B vs FW-F). (A) PCA score plot. (B) Volcano plot of differential features. (C) Permutation test for OPLS-DA model validation. (D) HCA heatmap based on the differential-feature set selected for the FW-B vs FW-F comparison. Note: 1. (A) The first two principal components collectively explain 64.02% of the total variance across all variables. 2. (C) Model validity was assessed by permutation testing (*n* = 200), yielding a negative Q^2^ intercept (-0.465). R^2^X (cum) = 0.406, R^2^Y (cum) = 0.931, Q^2^ (cum) = 0.924, CV-ANOVA *p* = 1.073 × 10^-9^.

**Fig. 4:**
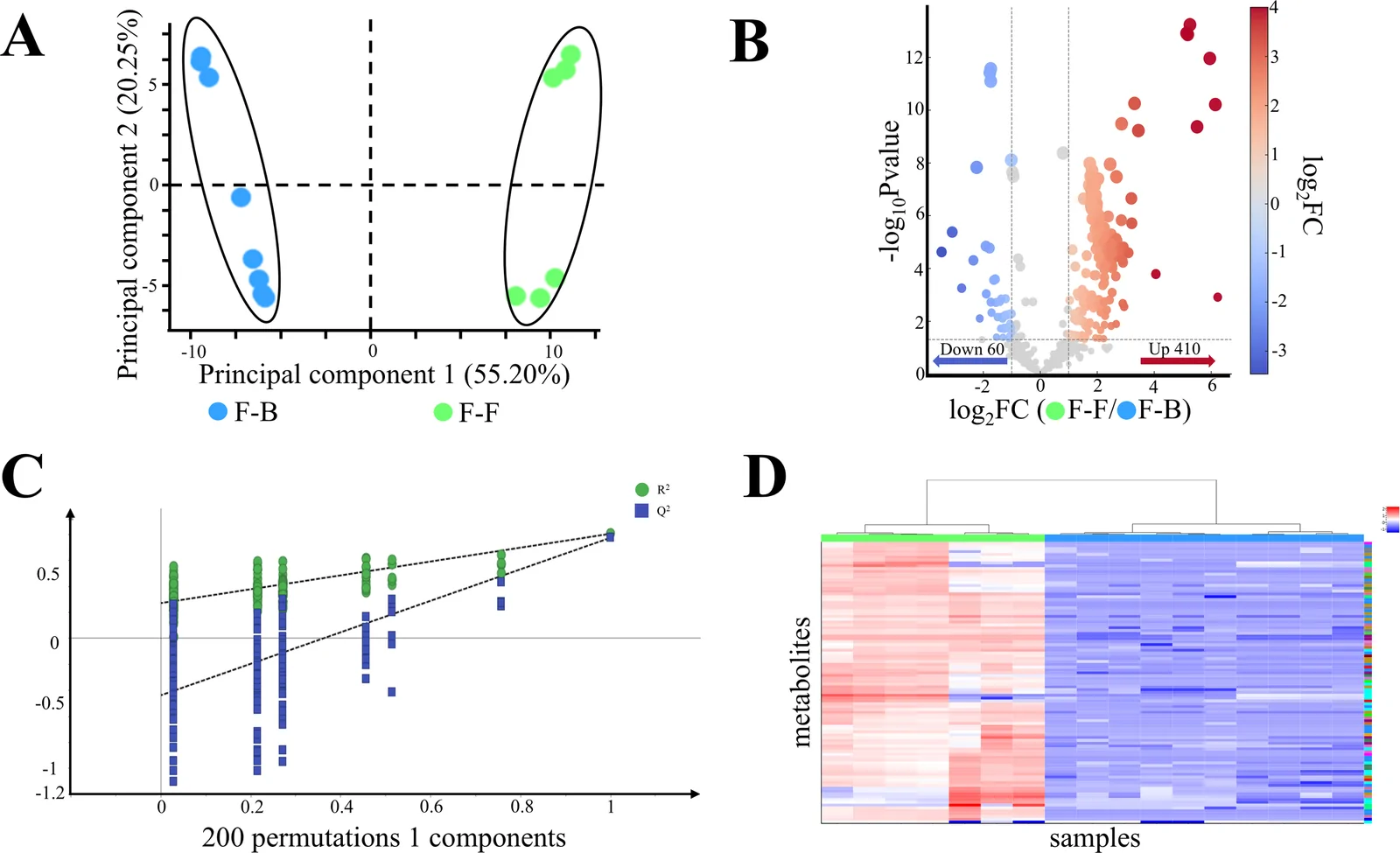
Metabolomic differences between bulbs and flowers of cultivated F (F-B vs F-F). (A) PCA score plot. (B) Volcano plot of differential features. (C) Permutation test for OPLS-DA model validation. (D) HCA heatmap based on the differential-feature set selected for the F-B vs F-F comparison. Note: 1. (A) The first two principal components collectively explain 75.45% of the total variance across all variables. 2. (C) Model validity was assessed by permutation testing (*n* = 200), yielding a negative Q^2^ intercept (-0.437). R^2^X (cum) = 0.933, R^2^Y (cum) = 0.806, Q^2^ (cum) = 0.775, CV-ANOVA *p* = 0.0079.

**Fig. 5:**
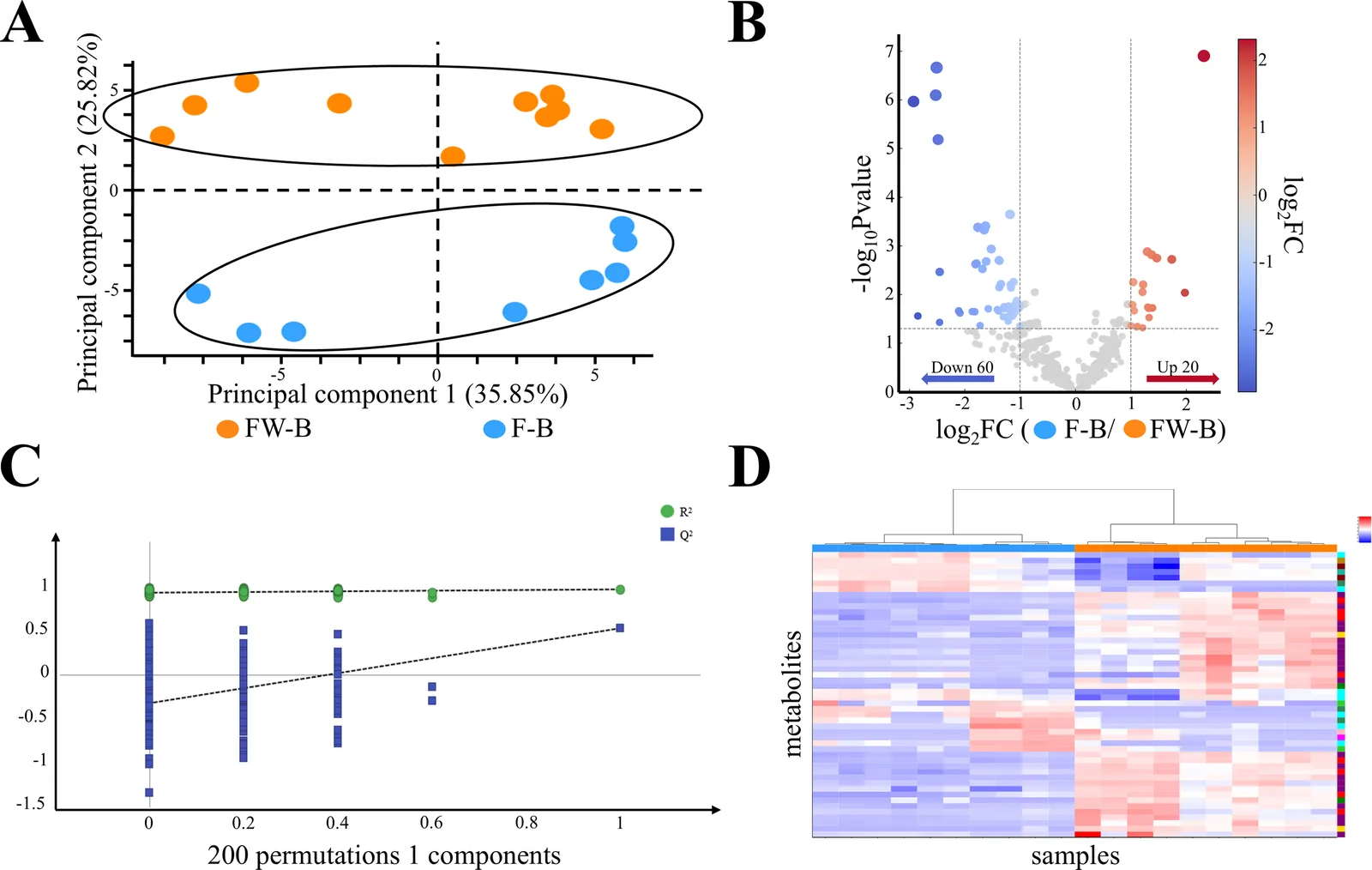
Metabolomic differences between bulbs of cultivated FW and F (FW-B vs F-B). (A) PCA score plot. (B) Volcano plot of differential features. (C) Permutation test for OPLS-DA model validation. (D) HCA heatmap based on the differential-feature set selected for the FW-B vs F-B comparison. Note: 1. (A) The first two principal components collectively explain 61.67% of the total variance across all variables. 2. (C) Model validity was assessed by permutation testing (*n* = 200), yielding a negative Q^2^ intercept (-0.314). R^2^X (cum) = 0.618, R^2^Y (cum) = 0.965, Q^2^ (cum) = 0.528, CV-ANOVA *p* = 0.15.

**Fig. 6:**
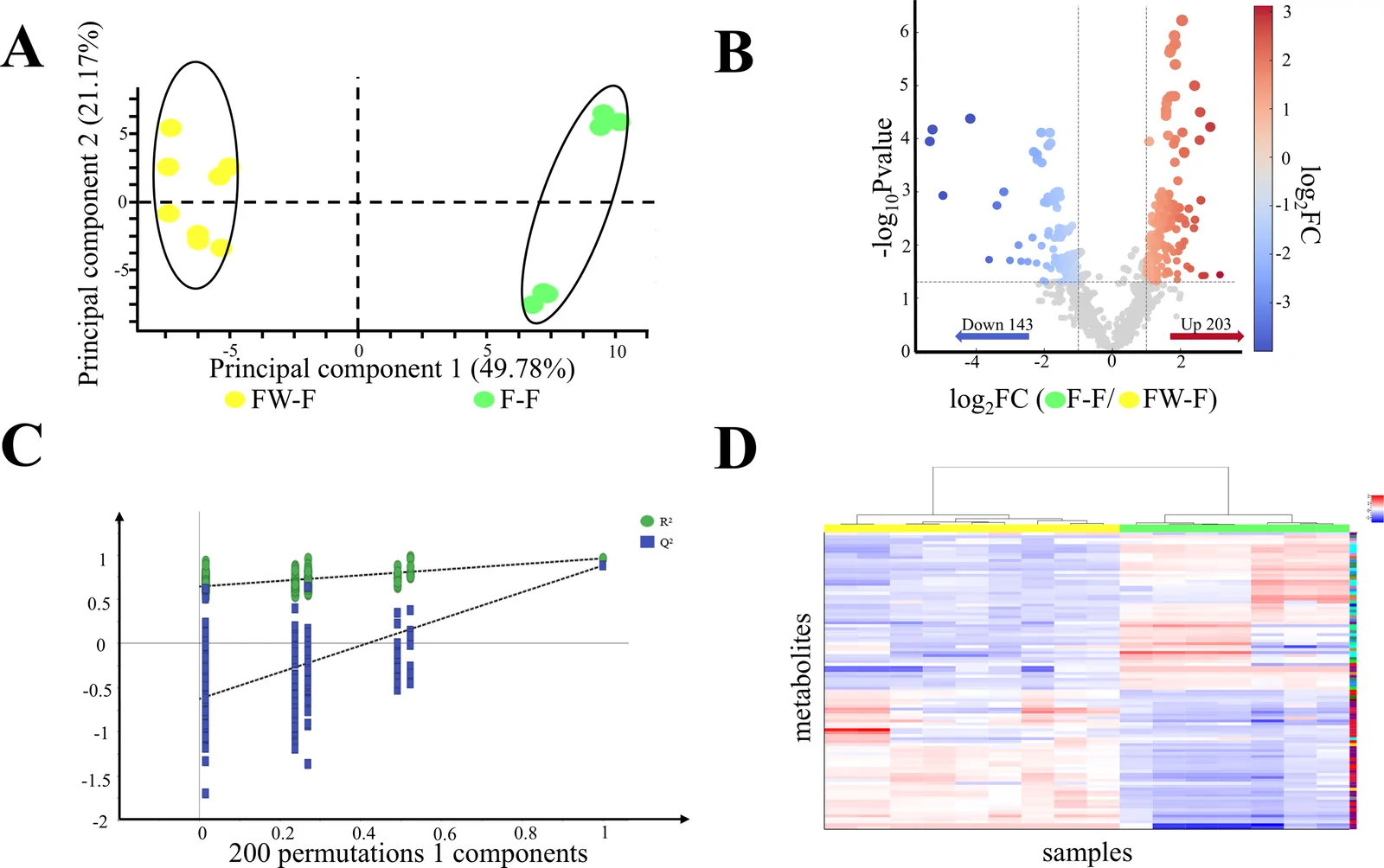
Metabolomic differences between flowers of cultivated FW and F (FW-F vs F-F). (A) PCA score plot. (B) Volcano plot of differential features. (C) Permutation test for OPLS-DA model validation. (D) HCA heatmap based on the differential-feature set selected for the FW-F vs F-F comparison. Note: 1. (A) The first two principal components collectively explain 70.95% of the total variance across all variables. 2. (C) Model validity was assessed by permutation testing (*n* = 200), yielding a negative Q^2^ intercept (-0.626). R^2^X (cum) = 0.709, R^2^Y (cum) = 0.966, Q^2^ (cum) = 0.886, CV-ANOVA *p* = 0.0008.

#### 3.2.2 Selection and identification of representative differential compounds

Candidate compounds were manually prioritized by integrating OPLS-DA VIP values (VIP > 1) and volcano-plot criteria (|log_2_FC| ≥ 1 and *p* < 0.05) with peak quality (peak shape and signal-to-noise ratio). Annotations were assigned using accurate mass, isotope patterns, and characteristic MS/MS fragmentation, and were confirmed with authentic standards (matching retention times and MS/MS spectra) when available; otherwise, putative annotations were supported by literature-consistent fragmentation evidence with manual curation, ensuring a traceable and evidence-driven chemical basis.

Differential analysis identified preliminary candidate compositional marker panels for both cultivated source and tissue (**Tables S8-S11**): 6 candidates for FW-B vs. FW-F (4 bulb-enriched and 2 flower-enriched), 7 candidates for F-B vs. F-F (2 bulb-enriched and 5 flower-enriched), 5 candidates for FW-B vs. F-B (1 FW-enriched and 4 F-enriched), and 4 candidates for FW-F vs. F-F (2 FW-enriched and 2 F-enriched). Representative candidates included arginine, sipeimine-3β-D-glucoside, and edpetiline or isomer for FW-B vs. FW-F; L-isoleucine, hupeheninoside, isorhamnetin, and fructosyl phenylalanine for F-B vs. F-F; edpetiline or isomer, puqienine E, hupeheninoside, β-solanine or isomer, and solasonine for FW-B vs. F-B; and uracil, L-isoleucine, β-solanine or isomer, and fructosyl isoleucine for FW-F vs. F-F. Compounds appearing in more than one pairwise comparison were considered overlapping candidate markers reflecting shared tissue- or cultivated-source-related differences, rather than as independent evidence from unrelated comparisons. Although no single metabolite was completely exclusive to one tissue or cultivated source across all 36 batches, these manually curated differential compounds provide a preliminary compositional marker panel for QC-oriented screening and future targeted validation. Because the FW-B versus F-B OPLS-DA model did not reach statistical significance by CV-ANOVA, the five candidates from this bulb-to-bulb comparison should be regarded as exploratory features requiring further validation.

### 3.3 Alkaloid quantification

Compositional and analytical studies have established steroidal alkaloids as characteristic constituents of *Fritillaria*, and sipeimine is listed in the Chinese Pharmacopoeia as a quantitative marker for quality control. However, single-marker practice may be insufficient for evaluating source/tissue differences and by-product materials under food-relevant contexts. Building on the candidate markers identified by untargeted metabolomics (**Tables S8-S11**, with alkaloids forming precisely half), targeted quantification of five representative alkaloids was performed across the 36 batches to validate their distribution patterns between sources and tissues.

Quantification results for the five alkaloids, together with statistical summaries, are provided in **Table S12**, and representative TIC chromatograms (**Fig. S12**) and MRM chromatograms (**Fig. S13**) of samples and mixed standards are shown in the Supplementary Material. To visualize between-group distributions, box plots were generated based on **Table S12** (Fig. 7), highlighting both central tendency and dispersion across the 36 batches.

**Fig. 7:**
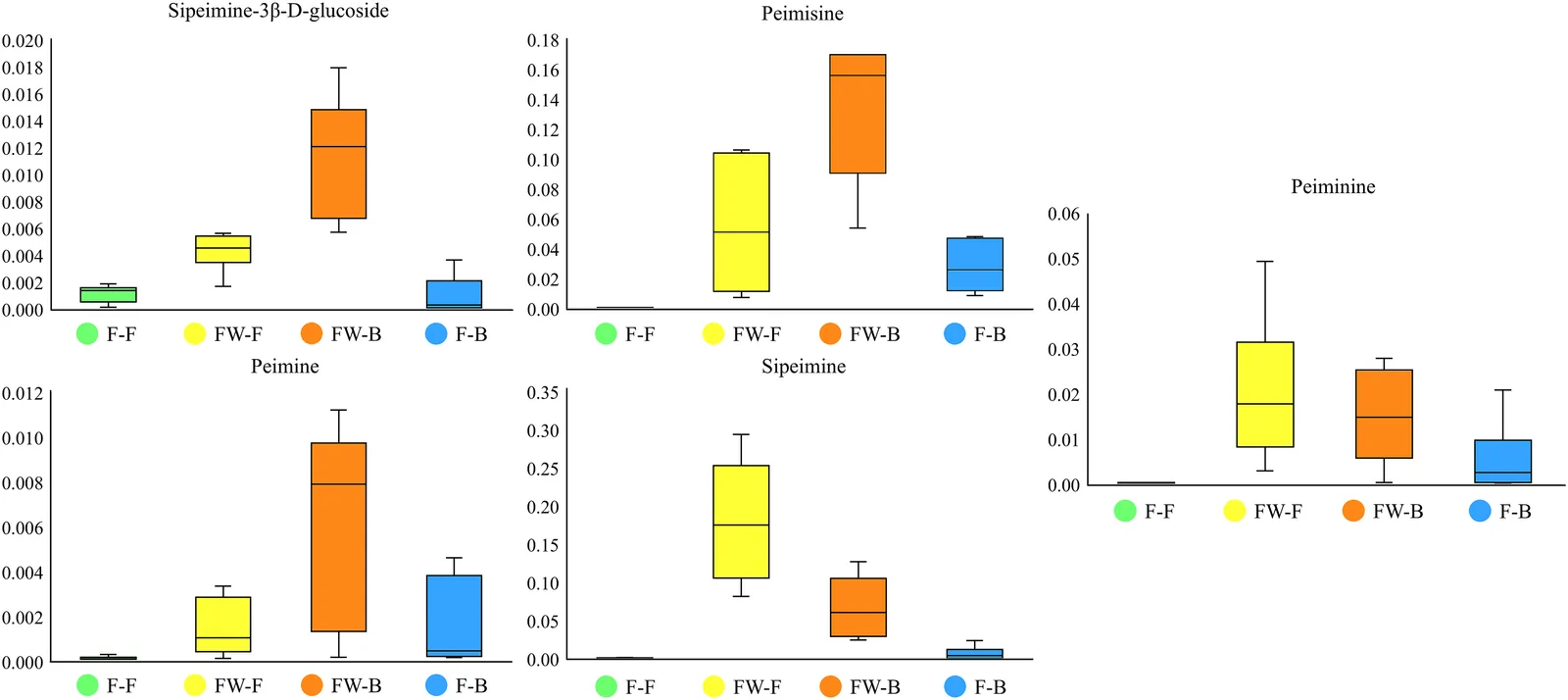
Box plots of five quantified alkaloids across four sample groups (units: mg/g, *n* = 36 batches). Detailed statistical results, including Kruskal-Wallis p values, ε^2^ effect-size estimates, and significant pairwise comparisons after Benjamini-Hochberg correction, are provided in **Table S12**.

All five alkaloids were detectable in both cultivated sources and in both tissues, but their abundance profiles differed markedly among groups, with significant overall group-dependent differences for all five targeted alkaloids based on Kruskal-Wallis tests (**Table S12**). Dunn's post hoc tests with Benjamini-Hochberg correction further showed that peimisine was significantly higher in FW-B than in FW-F (q = 0.0376), whereas sipeimine showed a higher distributional tendency in FW-F but did not retain a significant FW-F vs. FW-B pairwise difference after correction. Overall, alkaloid levels in FW-B tended to exceed those in F-B, with sipeimine-3β-D-glucoside and peimisine showing particularly evident enrichment in FW-B. Therefore, the alkaloid differences in FW flowers should be interpreted as a cautionary compositional signal rather than direct evidence of either food-use suitability or safety risk. These results indicate that flower by-products should not be regarded as simple substitutes for bulbs, and that alkaloid abundance should be considered in future food-related evaluation, particularly in relation to infusion-transfer behavior, sensory bitterness, bioaccessibility, toxicological relevance, and exposure assessment.

### 3.4 Molecular weight characterization of crude polysaccharides by GPC-RI-MALS

To characterize differences at the macromolecular level, GPC-RI-MALS was applied to profile molecular-weight distributions of crude polysaccharides from the 36 batches. Key molecular-weight parameters (e.g., *M*_w_ and PDI) are summarized in **Table S13**, and *M*_w_-based box plots are shown in **Fig. S14**.

The results indicated measurable differences in crude-polysaccharide molecular-weight distribution across sources and tissues. On average, F-B showed higher Mw values, suggesting differences in the molecular-weight distribution of the crude polysaccharides. However, considerable within-group variation was also observed, especially for *Mw* values in bulb samples, where several F-B batches showed markedly higher *Mw* values than the other groups, whereas some FW-B batches showed relatively low *Mw* values (Table S13). This variation may reflect differences in cultivation source, batch heterogeneity, extraction behavior, and the heterogeneous nature of crude polysaccharide fractions. All four groups showed PDI values exceeding 2, indicating pronounced heterogeneity in the crude-polysaccharide systems. Flower samples showed higher mean PDI values than the corresponding bulbs (FW-F: 5.70 vs FW-B: 3.64; F-F: 5.21 vs F-B: 4.85), suggesting a broader molecular-weight distribution in flowers on average (Whitfield et al., 2019). A Kruskal-Wallis test indicated an overall difference in PDI among the four groups (*p* = 0.0091, ε2 = 0.267), with a significant pairwise difference observed between FW-F and FW-B after Benjamini-Hochberg correction (q = 0.0047; Table S13). The broader PDI distribution in flowers may reflect the more heterogeneous carbohydrate matrix of reproductive tissues, including differences in cell-wall composition, soluble polysaccharide fractions, and tissue-specific carbohydrate turnover relative to underground storage bulbs.

### 3.5 Monosaccharide composition analysis of polysaccharides

After establishing molecular-weight differences in crude polysaccharides, we further examined compositional variation by quantifying 12 monosaccharides in the 36 batches using UHPLC-QQQ-MS: DL-Arabinose (Ara), D-(+)-Xylose (Xyl), D-Ribose (Rib), L-Fucose (Fuc), L-(+)-Rhamnose (Rha), D-(+)-Sorbose (Sor), D-Fructose (Fru), D-Galactose (Gal), D-Mannose (Man), D-(+)-Glucose (Glc), D-Galacturonic acid (GalA), and D-Glucuronic acid (GlcA). This panel was selected to cover common neutral, acidic, and deoxy monosaccharides relevant to plant polysaccharide composition, thereby providing broader compositional coverage than previously reported limited monosaccharide panels in Fritillaria polysaccharide studies. Detailed quantitative and statistical results, expressed as mg/g starting material, are summarized in **Table S14**, and corresponding box plots are shown in Fig. 8. Sor was not detected in any of the 36 batches under the present UHPLC-QQQ-MS method. Fru and Rib showed no significant overall differences among the four groups, whereas most other monosaccharides showed significant group-dependent differences. Overall, multiple monosaccharides (Rha, Man, Ara, GlcA, Gal, GalA, Fuc, and Xyl) displayed higher levels or enrichment trends in flower samples, whereas Glc was generally higher in bulb samples (Jiang et al., 2024). These findings indicate group-dependent differences in extractable polysaccharide-derived monosaccharide contents expressed per gram of starting material. However, because crude polysaccharide extraction yields were not separately determined, the observed differences should be interpreted as integrated changes in the extracted crude polysaccharides rather than as isolated evidence of total crude polysaccharide yield. Taken together with the molecular-weight distribution differences described above, these tissue-specific monosaccharide profiles provide complementary crude-polysaccharide-related compositional information for QC-oriented differentiation in processed formats where morphology is not informative.

**Fig. 8:**
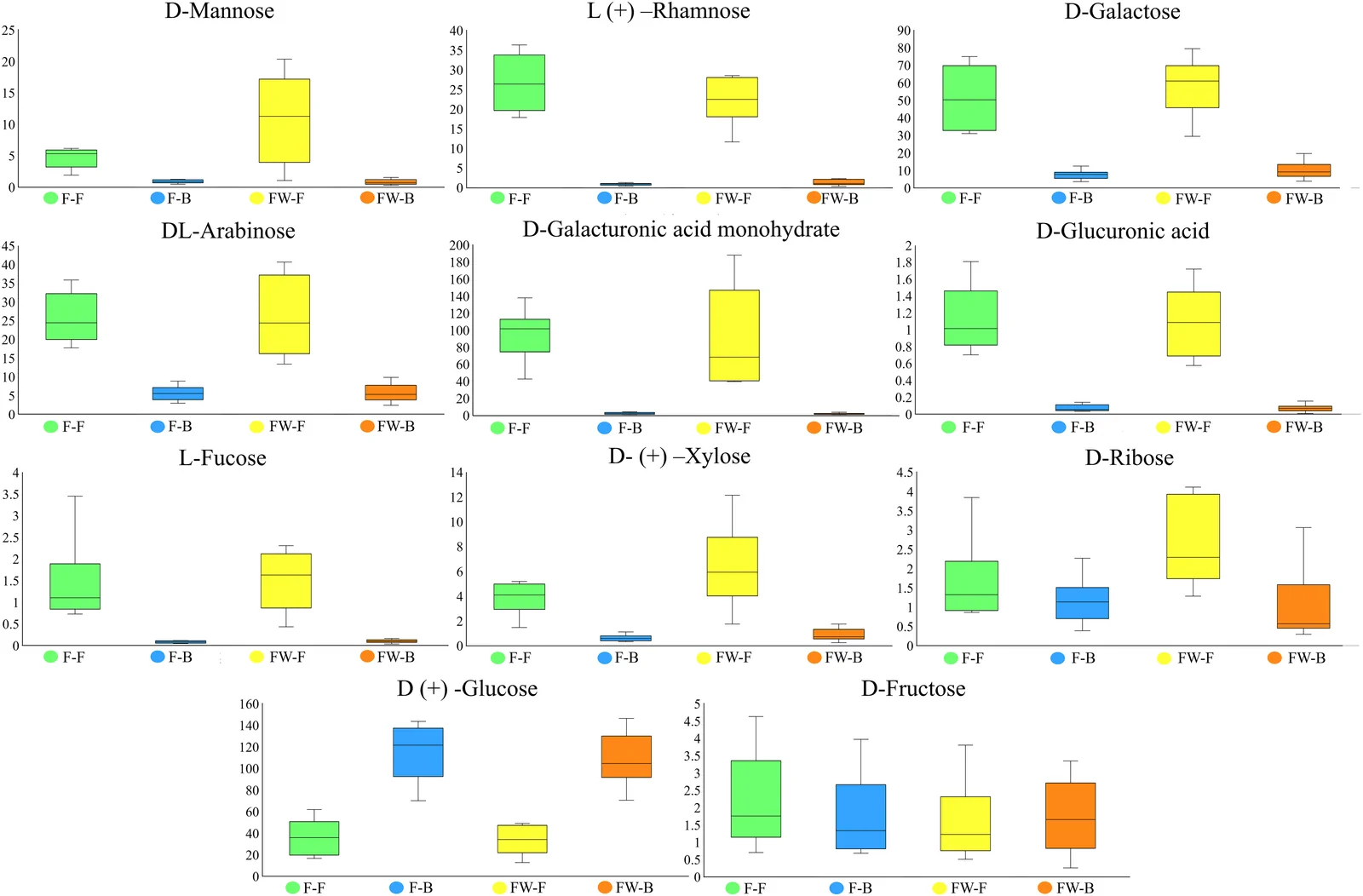
Box plots of quantified monosaccharides in polysaccharide hydrolysates across four sample groups (mg/g). Detailed statistical results, including Kruskal-Wallis p values, ε^2^ effect-size estimates, and significant pairwise comparisons after Benjamini-Hochberg correction, are provided in **Table S14**. Note: Sorbose was not detected and is not displayed.

### 3.6 Discussion

This study provides an integrated, QC-oriented compositional comparison of two dominant cultivated FCB sources (FW vs. F) across bulbs and flowers, combining small-molecule metabolomics with crude polysaccharide-related compositional profiling to support source/tissue discrimination and to provide a compositional baseline for future evaluation of flower by-products. These compositional differences support raw-material identity screening and batch-consistency control, and help define safety-, sensory-, exposure-, and product-development-related questions that require targeted evaluation in future food-related studies.

At the small-molecule level, targeted quantification corroborated the distribution patterns suggested by untargeted metabolomics: the five representative alkaloids showed pronounced source- and tissue-dependent variability, and notably, in FW, peimisine was significantly higher in bulbs than in the corresponding flowers, whereas sipeimine showed a higher distributional tendency (Fig. 7). These tissue-dependent alkaloid differences may be related to organ-specific metabolic allocation in Fritillaria, as flowers and bulbs differ in developmental function, defense-related chemical partitioning, and storage-associated metabolism. Considered alongside the differential compound lineages captured by untargeted screening and supported by manual annotation, these results suggest that both "type differences" and "tissue differences" can be represented by a set of small-molecule candidate markers, providing a data-driven starting point for targeted verification and QC-oriented evaluation (Jiang et al., 2024, Liu et al., 2020, Mialon, Roig, Capodanno and Cadiere, 2023, Wu et al., 2022).

From the perspective of future by-product evaluation, the observed organ-dependent alkaloid burden and broader metabolite differences imply that flowers should not be treated as a simple surrogate for bulbs (Li et al., 2022). As a reproductive organ, flowers may differ systematically from bulbs in development- and defense-associated metabolism, leading to organ-specific partitioning of alkaloids and other metabolites (Kumar et al., 2020; Lu et al., 2022). This organ dependence is also consistent with the tissue differences observed at the polysaccharide level (*Mw* distribution and expanded monosaccharide fingerprints), suggesting distinct compositional features of flowers across both small- and macromolecular layers (Borjigin et al., 2023). In particular, future studies under realistic preparation conditions (e.g., infusion) are warranted to evaluate infusion-transfer behavior, sensory properties, bioaccessibility, and preparation-specific exposure to pertinent alkaloids, and to relate the raw-material compositional differences observed here to processing-relevant attributes such as extraction behavior or viscosity-related properties (Kaltner et al., 2025; Luo et al., 2025). Although prepared infusions were not directly analyzed here, the raw-material alkaloid profiles identify peiminine, sipeimine, and related steroidal alkaloids as priority analytes for subsequent infusion-transfer and serving-size-based exposure assessment under realistic preparation conditions. These unresolved infusion-transfer-, preparation-, sensory-, bioaccessibility-, toxicological-, and exposure-related questions should be addressed before the alkaloid differences observed in dried materials are used to infer dietary safety, sensory acceptability, or acceptable intake levels.

At the macromolecular level, the crude-polysaccharide molecular-weight distribution and monosaccharide profiles provided information complementary to the alkaloid-centered small-molecule data. These differences may reflect tissue-specific carbohydrate allocation and cell-wall-related compositional variation between reproductive flower tissues and underground storage bulbs (Zhao et al., 2018), and may inform future evaluation of extraction behavior, processing consistency, and crude-polysaccharide compositional quality. Therefore, integrating "small-molecule metabolome + crude-polysaccharide molecular-weight distribution + monosaccharide composition" provides a broader multidimensional compositional basis for QC-oriented evaluation than reliance on a single alkaloid index alone (Zhao et al., 2025). To further connect these analytical layers, a Spearman correlation analysis was performed across the 36 matched batches (Fig. S15). PDI, but not *Mw*, was significantly associated with several monosaccharide variables after Benjamini-Hochberg correction, including Ara, GalA, GlcA, and Xyl, suggesting that molecular-weight dispersity was partly aligned with crude-polysaccharide compositional variation. In contrast, significant alkaloid–monosaccharide associations were limited to a small number of pairs, such as Peimisine-Glc and Peimine-GalA. These patterns indicate cross-layer consistency within the crude-polysaccharide dimension while supporting the complementary, rather than redundant, value of the small-molecule and crude-polysaccharide datasets for QC-oriented differentiation.

Several priorities for future work remain. Although the present sample set covered five cultivation bases and was collected within the same harvest period under a standardized drying/storage workflow, future validation using larger and more balanced cohorts with more detailed agronomic and environmental metadata would further strengthen the generalizability of the proposed candidate marker panel. Because crude polysaccharide extraction yields were not separately determined across all batches, whether extraction yield itself differed among groups remains unknown from the current data. Accordingly, the GPC-RI-MALS-derived *Mw* and PDI values are used as comparative molecular-weight distribution descriptors of standardized crude-polysaccharide fractions, rather than as fine structural parameters of purified polysaccharides, such as glycosidic linkages, branching degree, FTIR/NMR features, methylation-derived linkage patterns, or conformational properties. Because a fixed dn/dc value was applied to all samples, compositional differences among crude-polysaccharide fractions may influence absolute *Mw* estimates, but the identical calculation condition supports within-study comparison of molecular-weight distribution patterns. Therefore, the monosaccharide data should be interpreted as extractable polysaccharide-derived monosaccharide contents under the standardized workflow, rather than as direct measurements of total crude polysaccharide yield. The preliminary candidate compositional marker panel requires verification in larger, independently sourced cohorts, ideally including 20–30 independent batches per group. Priority should be given to Level 1 or quantitatively confirmed compounds with relatively stable statistical support, such as peiminine, peimisine, peimine, sipeimine-3β-D-glucoside, and recurrent alkaloid-related candidates from Tables **S8–S11**. Linking these chemical markers to food-related properties, including infusion-transfer behavior, antioxidant capacity, and processing-related traits, would further strengthen the evidence base for future food-related evaluation. Overall, this work provides interpretable chemical evidence for differentiating bulbs and flowers of cultivated FW and F and offers a compositional basis for future QC-oriented and food-related evaluation of cultivation by-products (Bian et al., 2025). Future studies using stable isotope-labelled internal standards and/or matrix-matched calibration would further improve matrix-effect correction for targeted UHPLC-QQQ-MS quantification, particularly when rigorous absolute quantification or exposure assessment is required. Systematic incorporation of negative-ion-mode data would also improve coverage of acidic and other negative-ion-responsive metabolites. Future integration of chemical profiling with DNA fingerprinting or other molecular authentication approaches may further improve taxonomic resolution and authentication reliability.

## 4 Conclusions

In summary, this study provides a compositional basis for profiling and evaluating the quality consistency of cultivated FCB botanical materials relevant to future food-related evaluation. Using UHPLC-Q-TOF-MS, UHPLC-QQQ-MS, and GPC-RI-MALS, we compared flowers and bulbs from cultivated FW and F at both the small-molecule and polysaccharide levels. A total of 76 compounds were annotated, and differential features identified by metabolomics were refined into representative compounds through library matching and manual verification. We also established and validated a method for the simultaneous quantification of 12 monosaccharides, which enabled polysaccharide comparison across tissues and cultivated sources. The preliminary marker candidates identified here may serve as useful references for distinguishing flowers from bulbs and for evaluating material consistency, pending further validation in independent cohorts. Notably, FW-F flowers differed from the corresponding bulbs in targeted alkaloid distribution patterns, while flower samples showed elevated polysaccharide-derived monosaccharides such as Ara, GalA, GlcA, and Xyl, indicating that flowers should be considered separately as compositionally distinct cultivation by-products in future food-related evaluation. Overall, these results offer a compositional baseline for future safety, sensory, processing, infusion-transfer, and exposure-related studies, which should be further supported by independent validation of the proposed markers under relevant processing or infusion conditions.

## CRediT authorship contribution statement

**Zhao Geng:** Conceptualization, Methodology, Funding acquisition, Writing – review & editing, Supervision. **Yijie Ren:** Investigation, Formal analysis, Data curation, Writing – original draft. **Zhen Hu:** Formal analysis. **Chengjun He:** Data curation, Formal analysis, Writing – original draft, Writing – review & editing. **Donglin Gu:** Investigation. **Zhichun Zhang:** Methodology. **Yan Gou:** Methodology, Project administration, Supervision, Writing – review & editing. **Jingzhe Pu:** Formal analysis. **Yazhong Zhang:** Supervision, Writing – review & editing. **Lei Yang:** Formal analysis, Validation, Writing – review & editing. **Jincai Lu:** Methodology, Validation, Writing – review & editing.

## Declaration of competing interest

The authors declare that they have no known competing financial interests or personal relationships that could have appeared to influence the work reported in this paper.

## Data Availability

Data supporting the findings of this study are provided in the Supplementary Material or are available from the corresponding author upon reasonable request.
