# Supplementary material for: Metabolomics and polysaccharide fingerprinting reveal compositional differences between cultivated Fritillaria bulbs and flower by-products
Source: Food Chem X. 2026 Aug 21;39:104348. doi: 10.1016/j.fochx.2026.104348 (PMC13671043; doi:10.1016/j.fochx.2026.104348)
Supplement: Supplementary file 1 [file mmc1.docx]

**Supplementary data**

**Metabolomics and polysaccharide fingerprinting reveal compositional differences between cultivated *Fritillaria* bulbs and flower by-products**

Zhao Geng^a,1,*^, Yijie Ren^a,c,[1]^, Zhen Hu^c^, Chengjun He^a,^, Donglin Gu^a^, Zhichun Zhang^a^, Yan Gou^a,*^, Jingzhe Pu^b^, Yazhong Zhang^b,*^ , Lei Yang^a^, Jincai Lu^d^

a Sichuan Institute for Drug Control (Sichuan Testing Center of Medical Devices, Sichuan Institute of Musk Deer Breeding), Chengdu 611731, China

b Anhui Institute for Food and Drug Control (NMPA Key Laboratory for Quality Research and Evaluation of Traditional Chinese Medicine), Hefei 230051, China

c Sichuan Key Laboratory of Fruit and Vegetable Postharvest Physiology, College of Food Science, Sichuan Agricultural University, Ya'an 625014, China

d School of Traditional Chinese Materia Medica, Shenyang Pharmaceutical University, Shenyang 110016, China

**Contents**

[Table S1. Sample information table	2](#_Toc221558850)

[Table S2. Reference standard information table	2](#_Toc221558851)

[Fig. S1. TIC plots of different solvents, mobile phase compositions, and columns	4](#_Toc221558852)

[Fig. S2. TIC chromatograms in positive-ion mode: F-F (A), FW-F (B), FW-B (C), and F-B (D)	5](#_Toc221558853)

[Fig. S3. TIC chromatograms in negative-ion mode: F-F (A), FW-F (B), FW-B (C), and F-B (D)	5](#_Toc221558854)

[Fig. S4. TIC chromatograms of five mobile phase combinations	6](#_Toc221558855)

[Fig. S5. TIC chromatograms of ACQUITY UPLC® BEH C18 (2.1 mm × 150 mm, 1.7 μm) (A), CAPCELL PAK ADME (2.1 mm × 100 mm, 2 μm) (B), and ACQUITY UPLC® BEH C18 (2.1 mm × 100 mm, 1.7 μm) (C)	6](#_Toc221558856)

[Fig. S6. Comparison of TIC chromatograms between the standard (A) and sample B-5 (B)	7](#_Toc221558857)

[Fig. S7. MRM chromatograms of 12 monosaccharides	7](#_Toc221558858)

[Table S3. Chemical composition selected by quality control (QC) and the relevant results of QC in positive-ion mode (n = 20)	8](#_Toc221558859)

[Table S4. Optimized MRM parameters	9](#_Toc221558860)

[Table S5. Method validation data for 12 monosaccharides	10](#_Toc221558861)

[Table S6. Current-batch confirmation data for five alkaloids	11](#_Toc221558862)

[Table S7. Results of UHPLC-Q-TOF-MS analyses	12](#_Toc221558863)

[Fig. S8. Fragmentation pattern of compound 29	19](#_Toc221558864)

[Fig. S9. Fragmentation pattern of compound 40	19](#_Toc221558865)

[Fig. S10. Fragmentation pattern of compound 17	20](#_Toc221558866)

[Fig. S11. Fragmentation pattern of compound 19	20](#_Toc221558867)

[Table S8. Representative differential compounds between FW-B and FW-F	21](#_Toc221558868)

[Table S9. Representative differential compounds between F-B and F-F	21](#_Toc221558869)

[Table S10. Representative differential compounds between FW-B and F-B	22](#_Toc221558870)

[Table S11. Representative differential compounds between FW-F and F-F	22](#_Toc221558871)

[Fig. S12. TIC chromatograms of B-5 (A) and STD (mixed reference standards) (B)	23](#_Toc221558872)

[Fig. S13. MRM chromatograms of 5 alkaloids	23](#_Toc221558873)

[Table S12. Quantitative results of 36 batches using UHPLC-QQQ-MS (mg/g)	23](#_Toc221558874)

[Table S13. GPC-RI-MALS analysis results of 36 batches of samples	25](#_Toc221558875)

[Fig. S14. Box plot of *M*_w_ across 36 batches of samples	26](#_Toc221558876)

[Table S14. UHPLC-QQQ-MS analysis results of 12 monosaccharides	27](#_Toc221558877)

[Fig. S15. Spearman correlation heatmap between targeted alkaloids, GPC-RI-MALS-derived parameters, and quantifiable polysaccharide-derived monosaccharides across 36 Fritillaria batches	30](#_Toc221558877)

[References	31](#_Toc221558878)

# **Table S1.** Sample information table

| Sample ID | Sample Name | Source | Sample ID | Sample Name | Source |
| --- | --- | --- | --- | --- | --- |
| F-1 | F-F | SH | B-3 | F-B | SH |
| F-2 | F-F | SH | B-4 | FW-B | DFT |
| F-3 | F-F | SH | B-5 | FW-B | DFT |
| F-4 | FW-F | DFT | B-6 | F-B | SH |
| F-5 | FW-F | GQ | B-7 | F-B | SH |
| F-6 | FW-F | SZXB | B-8 | F-B | SH |
| F-7 | F-F | SZXB | SB-9 | FW-B | SZXB |
| F-8 | FW-F | SZXB | SB-10 | F-B | TQ |
| F-9 | F-F | SZXB | SB-11 | FW-B | TQ |
| F-10 | FW-F | SZXB | SB-12 | FW-B | TQ |
| F-11 | FW-F | DFT | B-13 | FW-B | TQ |
| SF-12 | FW-F | SZXB | B-14 | FW-B | TQ |
| SF-13 | F-F | TQ | B-15 | FW-B | TQ |
| SF-14 | FW-F | TQ | B-16 | FW-B | TQ |
| SF-15 | FW-F | TQ | B-17 | F-B | TQ |
| F-16 | F-F | SH | B-18 | F-B | TQ |
| B-1 | FW-B | DFT | B-19 | F-B | TQ |
| B-2 | F-B | SH | B-20 | F-B | TQ |

Note: 1. Samples were collected from: Ngapa County, Shenhe Agricultural Development Co., Ltd. (SH); Heishui County Sichuan Difeite Pharmaceutical Co., Ltd. (DFT); Sichuan Guoqing Fritillaria Biological Technology Co., Ltd. (GQ); Songpan County, Sichuan Songzan Xuebei Biotechnology Co., Ltd. (SZXB); Shangri-la, Tianquan Chuanbei Technology Co., Ltd. (TQ). 2. *Fritillaria unibracteata* Hsiao et K.C.Hsia var. *wabuensis* (FW; bulb: FW-B; flower: FW-F), *Fritillaria unibracteata* Hsiao et K.C.Hsia (F; bulb: F-B; flower: F-F).

# **Table S2.** Reference standard information table

| NO. | Name | Source | Batch | CAS | Purity |
| --- | --- | --- | --- | --- | --- |
| **Reference standards used in UHPLC-Q-TOF-MS experiments** | | | | | |
| 1 | Peimine | National Institutes for Food and Drug Control | 110750-201913 | 23496-41-5 | 98.4% |
| 2 | Peiminine | National Institutes for Food and Drug Control | 110751-202213 | 18059-10-4 | 98.3% |
| 3 | Adenosine | National Institutes for Food and Drug Control | 110879-202204 | 58-61-7 | 99.4% |
| 4 | Protocatechuic acid | National Institutes for Food and Drug Control | 110809-201906 | 99-50-3 | 97.7% |
| 5 | Caffeic acid | National Institutes for Food and Drug Control | 110885-201703 | 331-39-5 | 99.7% |
| 6 | Ferulic acid | National Institutes for Food and Drug Control | 110773-201915 | 537-98-4 | 99.4% |
| 7 | Proline | National Institutes for Food and Drug Control | 140677-202109 | 147-85-3 | 100.0% |
| 8 | Narcissoside | National Institutes for Food and Drug Control | 111977-201501 | 604-80-8 | 93.1% |
| 9 | Sipeimine | National Institutes for Food and Drug Control | 110767-202111 | 61825-98-7 | 99.4% |
| 10 | Aesculetin | National Institutes for Food and Drug Control | 110741-202109 | 305-01-1 | 96.0% |
| 11 | Arginine | National Institutes for Food and Drug Control | 140685-202108 | 74-79-3 | 100.0% |
| 12 | Uracil | National Institutes for Food and Drug Control | 100469-201302 | 66-22-8 | 99.6% |
| 13 | Peimisine | Chengdu Must Bio-Technology Co., Ltd. | MUST-23032815 | 19773-24-1 | 99.60% |
| 14 | Leucine | National Institutes for Food and Drug Control | 110876-202206 | 61-90-5 | 99.9% |
| 15 | Quercetin | National Institutes for Food and Drug Control | 100081-201610 | 117-39-5 | 99.8% |
| 16 | Nicotinic acid | National Institutes for Food and Drug Control | 100434-201102 | 59-67-6 | 99.9% |
| 17 | Luteolin | National Institutes for Food and Drug Control | 111520-202006 | 491-70-3 | 94.4% |
| 18 | Sipeimine-3β-D-glucoside | National Institutes for Food and Drug Control | 111917-201202 | 32685-93-1 | 96.4% |
| **Reference standards used in GPC-RI-MALS experiments** | | | | | |
| 1 | Dextran Standards D5 Mp36800 | National Institutes for Food and Drug Control | 140637-201203 | 9004-54-0 | / |
| **Monosaccharide reference standards used in UHPLC-QQQ-MS experiments** | | | | | |
| 1 | DL-Arabinose | Chengdu Must Bio-Technology Co., Ltd. | MUST-23022719 | 147-81-9 | 98.25% |
| 2 | D- (+) -Xylose | Chengdu Must Bio-Technology Co., Ltd. | MUST-23111701 | 58-86-6 | 99.59% |
| 3 | D-Ribose | Chengdu Must Bio-Technology Co., Ltd. | MUST-24041327 | 50-69-1 | 98.00% |
| 4 | L-Fucose | Chengdu Must Bio-Technology Co., Ltd. | MUST-23050615 | 2438-80-4 | 99.32% |
| 5 | L- (+) -Rhamnose | Chengdu Must Bio-Technology Co., Ltd. | MUST-23091415 | 10030-85-0 | 98.33% |
| 6 | D- (+) -Sorbose | Chengdu Must Bio-Technology Co., Ltd. | MUST-24051620 | 3615-56-3 | 99.95% |
| 7 | D-Glucuronic acid | Chengdu Must Bio-Technology Co., Ltd. | MUST-23021309 | 6556-12-3 | 98.00% |
| 8 | D-Galactose | Chengdu Must Bio-Technology Co., Ltd. | MUST-23111114 | 59-23-4 | 99.51% |
| 9 | D-Mannose | Chengdu Must Bio-Technology Co., Ltd. | MUST-24022822 | 3458-28-4 | 99.52% |
| 10 | D (+) -Glucose | Chengdu Must Bio-Technology Co., Ltd. | MUST-23030214 | 50-99-7 | 99.61% |
| 11 | D-Galacturonic acid | Chengdu Must Bio-Technology Co., Ltd. | MUST-23021308 | 91510-62-2 | 98.00% |
| 12 | D-Fructose | Chengdu Push Bio-Technology Co., Ltd. | PS020484 | 57-48-7 | 98.00% |
| **Alkaloid reference standards used in UHPLC-QQQ-MS experiments** | | | | | |
| Corresponding to chemical constituents No. 1, 2, 9, 13, and 18 listed in Table S2 (UHPLC-Q-TOF-MS reference standards). | | | | | |

Before chemical profiling, UHPLC-Q-TOF-MS conditions were optimized to maximize signal response and chromatographic resolution. Three aqueous phases were tested: 0.1% formic acid in water (A1), 10 mmol/L ammonium formate in water (A2), and 10 mmol/L ammonium formate with 0.1% formic acid (A3). Two organic phases—methanol (B1) and acetonitrile (B2)—were compared. Column performance was assessed using two columns: a Thermo Hypersil GOLD aQ (2.1 mm × 150 mm, 1.9 μm) and a Waters ACQUITY UPLC HSS T3 (2.1 mm × 100 mm, 1.8 μm). We also investigated five extraction solvents: 30% (v/v) methanol (C1), 50% (v/v) methanol (C2), pure methanol (C3), 80% (v/v) ethanol (C4), and 80% (v/v) methanol (C5).

# **Fig. S1.** TIC plots of different solvents, mobile phase compositions, and columns

Therefore, the Thermo Hypersil GOLD aQ column (2.1 × 150 mm, 1.9 μm), with 0.1% formic acid in water as the aqueous phase and methanol as the organic phase, in combination with 80% (v/v) methanol as the extraction solvent, was selected. This configuration delivered the strongest MS response, the largest number of detectable peaks, and improved separation under the tested optimization conditions (Fig. S1).

# **Fig. S2.** TIC chromatograms in positive-ion mode: F-F (A), FW-F (B), FW-B (C), and F-B (D)

# **Fig. S3.** TIC chromatograms in negative-ion mode: F-F (A), FW-F (B), FW-B (C), and F-B (D)

For monosaccharide composition analysis by UHPLC-QQQ-MS, the method was also optimized. Because monosaccharides are largely structural isomers and PMP (1-phenyl-3-methyl-5-pyrazolone) derivatization generates additional isomeric derivatives, simultaneous determination of multiple targets posed substantial chromatographic challenges. Building on published protocols for monosaccharide analysis, we evaluated several mobile phase systems. The aqueous phase was either 0.1% formic acid (containing 5 mmol/L ammonium acetate) or water, and the organic phase was methanol or acetonitrile. Accordingly, five elution systems were examined: 0.1% formic acid-methanol, 0.1% formic acid-acetonitrile, 0.1% formic acid (5 mmol/L ammonium acetate) -methanol, 0.1% formic acid (5 mmol/L ammonium acetate) -acetonitrile, and water-acetonitrile (Fig. S4). In parallel, three columns were compared, and the final column was selected based on overall resolution and signal intensity (Fig. S5).

# **Fig. S4.** TIC chromatograms of five mobile phase combinations: A (0.1% formic acid containing 5 mmol/L ammonium acetate-acetonitrile), B (0.1% formic acid-methanol), C (0.1% formic acid-acetonitrile), D (0.1% formic acid containing 5 mmol/L ammonium acetate-methanol), and E (water-acetonitrile)

# **Fig. S5.** TIC chromatograms of ACQUITY UPLC® BEH C18 (2.1 mm × 150 mm, 1.7 μm) (A), CAPCELL PAK ADME (2.1 mm × 100 mm, 2 μm) (B), and ACQUITY UPLC® BEH C18 (2.1 mm × 100 mm, 1.7 μm) (C)

As shown in Fig. S4, four elution systems (B, C, D, and E) failed to achieve effective separation within 41 min and instead produced a broad, unresolved envelope peak. Fig. S5 further showed that the peak shape and resolution obtained on columns B and C were suboptimal. Therefore, the ACQUITY UPLC® BEH C18 column (2.1 mm × 150 mm, 1.7 μm) was selected, using 0.1% formic acid containing 5 mmol/L ammonium acetate as the aqueous phase and acetonitrile as the organic phase (Fig. S6 and S7). Table S4 summarizes the optimized MRM parameters.

# **Fig. S6.** Comparison of TIC chromatograms between the standard (A) and sample B-5 (B)

# **Fig. S7.** MRM chromatograms of 12 monosaccharides

# **Table S3.** Chemical composition selected by quality control (QC) and the relevant results of QC in positive-ion mode (*n* = 20)

| NO. | Retention  time | Parent ion  *m/z* | Retention  time RSD% | *m/z*  RSD‰ | Area  RSD% | Mean RT RSD% | Mean *m/z*  RSD‰ | Mean area  RSD% | |
| --- | --- | --- | --- | --- | --- | --- | --- | --- | --- |
| 1 | 1.33 | 104.1069 | 0.12 | 0.01 | 2.67 | 0.06 | 0.02 | 4.19 |  |
| 2 | 1.83 | 280.1389 | 0.04 | 0.03 | 3.54 |  |  |  |  |
| 3 | 6.31 | 234.1333 | 0.08 | 0.01 | 4.12 |  |  |  |  |
| 4 | 9.04 | 789.2053 | 0.07 | 0.01 | 2.98 |  |  |  |  |
| 5 | 11.37 | 291.0975 | 0.01 | 0.02 | 6.75 |  |  |  |  |
| 6 | 14.60 | 307.1011 | 0.03 | 0.01 | 3.45 |  |  |  |  |
| 7 | 16.48 | 979.2138 | 0.05 | 0.02 | 5.33 |  |  |  |  |
| 8 | 19.21 | 453.7330 | 0.09 | 0.01 | 4.20 |  |  |  |  |
| 9 | 20.65 | 708.4705 | 0.10 | 0.03 | 7.15 |  |  |  |  |
| 10 | 21.54 | 344.2268 | 0.02 | 0.02 | 2.34 |  |  |  |  |
| 11 | 22.89 | 240.1582 | 0.03 | 0.05 | 4.67 |  |  |  |  |
| 12 | 26.52 | 724.4536 | 0.01 | 0.01 | 5.21 |  |  |  |  |
| 13 | 33.38 | 149.0592 | 0.05 | 0.03 | 3.89 |  |  |  |  |
| 14 | 34.27 | 365.2767 | 0.13 | 0.01 | 4.56 |  |  |  |  |
| 15 | 36.15 | 181.0851 | 0.06 | 0.02 | 5.12 |  |  |  |  |
| 16 | 37.10 | 403.2309 | 0.09 | 0.04 | 6.34 |  |  |  |  |
| 17 | 40.77 | 377.2663 | 0.05 | 0.01 | 3.20 |  |  |  |  |
| 18 | 41.76 | 518.3221 | 0.08 | 0.02 | 2.15 |  |  |  |  |
| 19 | 44.36 | 675.6775 | 0.01 | 0.01 | 7.89 |  |  |  |  |
| 20 | 47.27 | 722.5272 | 0.08 | 0.03 | 1.76 |  |  |  |  |

# **Table S4.** Optimized MRM parameters

| **NO.** | **Name** | **Retention time/min** | **Fragmentor/V** | **Parent ion**  ***m/z*** | **Qualifier ion**  ***m/z*** | **Collision energy/V** | **Quantifier ion**  ***m/z*** | **Collision energy/V** |
| --- | --- | --- | --- | --- | --- | --- | --- | --- |
| **MRM method for monosaccharide composition analysis of polysaccharides** | | | | | | | | |
| 1 | DL-Arabinose | 34.644 | 121 | 481.2 | 373.2 | 23 | 187.2 | 43 |
| 2 | D- (+) -Xylose | 35.377 | 172 | 481.2 | 373.1 | 25 | 175.1 | 35 |
| 3 | D-Ribose | 23.027 | 135 | 481.2 | 373.1 | 25 | 175.1 | 35 |
| 4 | L-Fucose | 36.760 | 115 | 495.2 | 285 | 20 | 373.2 | 28 |
| 5 | L- (+) -Rhamnose | 23.049 | 163 | 495.2 | 267 | 30 | 217.1 | 34 |
| 6 | D- (+) -Sorbose | 19.816 | 96 | 510.2 | 402.2 | 22 | 175.1 | 35 |
| 7 | D-Fructose | 19.340 | 140 | 510.3 | 404.2 | 25 | 175.1 | 30 |
| 8 | D-Galactose | 32.457 | 101 | 511.2 | 373.1 | 28 | 175.1 | 35 |
| 9 | D-Mannose | 19.772 | 135 | 511.2 | 373.1 | 25 | 175.1 | 34 |
| 10 | D (+) -Glucose | 31.247 | 110 | 511.2 | 373.1 | 26 | 217.1 | 34 |
| 11 | D-Galacturonic acid | 30.948 | 167 | 525.2 | 241.1 | 30 | 187.2 | 44 |
| 12 | D-Glucuronic acid | 29.959 | 92 | 525.2 | 217.1 | 35 | 175.1 | 35 |
| **MRM method for alkaloid analysis** | | | | | | | | |
| 13 | Sipeimine-3β-D-glucoside | 3.05 | 192 | 592.4 | 574.2 | 44 | 138.1 | 59 |
| 14 | Peimisine | 4.52 | 178 | 428.4 | 114.1 | 36 | 109.1 | 36 |
| 15 | Peimine | 5.32 | 148 | 432.4 | 414.2 | 40 | 77.1 | 136 |
| 16 | Peiminine | 6.48 | 148 | 430.3 | 412.2 | 40 | 91.1 | 112 |
| 17 | Sipeimine | 4.10 | 148 | 430.3 | 412.2 | 36 | 138 | 56 |

# **Table S5.** Method validation data for 12 monosaccharides

| NO. | Name | Regression equation | R^2^ | Linearity range (μg/mL) | Precision% | Solution stability RSD% (*n* = 7) | | | Repeatability  RSD% | | Recovery  (Mean%/RSD%) | | LOD  (mg/kg) | LOQ  (mg/kg) |
| --- | --- | --- | --- | --- | --- | --- | --- | --- | --- | --- | --- | --- | --- | --- |
|  |  |  |  |  |  | STD | F | B | F | B | F | B |  |  |
| 1 | Fru | Y=331891675.115976X+146819.5862 | 0.9949 | 3.2~819.1 | 1.35 | 5.96 | 6.01 | 3.21 | 0.80 | 1.21 | 102/2.92 | 94/4.19 | 0.0003 | 0.0097 |
| 2 | Man | Y=1445221552.701210X+5400882.800 | 0.9916 | 3.8~543.1 | 3.22 | 4.82 | 9.09 | 4.61 | 1.90 | 2.29 | 97/7.81 | 98/4.29 | 0.0033 | 0.0109 |
| 3 | Rha | Y=60354881.333478X+449597.762788 | 0.9911 | 4.1~957.3 | 3.40 | 6.04 | 5.91 | 5.96 | 4.34 | 3.67 | 91/4.94 | 94/7.24 | 0.0001 | 0.0002 |
| 4 | Rib | Y=38176614.773876X+225776.072759 | 0.9936 | 4.0~933.9 | 2.10 | 4.32 | 6.09 | 5.61 | 4.34 | 2.25 | 103/4.71 | 102/6.29 | 0.0029 | 0.0095 |
| 5 | GlcA | Y=453544342.711902X+83932.363767 | 0.9937 | 0.05~61.6 | 1.81 | 1.82 | 4.19 | 2.91 | 3.28 | 3.25 | 107/4.11 | 102/3.28 | 0.0001 | 0.0002 |
| 6 | Glc | Y=191521634.637232X+2265682.4985 | 0.9911 | 150~3000 | 3.10 | 3.51 | 3.96 | 4.69 | 3.50 | 2.19 | 96/4.76 | 95/3.91 | 0.0007 | 0.0026 |
| 7 | GalA | Y=478002.206299X+5299.553984 | 0.9999 | 5.1~3500 | 3.29 | 6.53 | 6.07 | 4.91 | 5.13 | 4.61 | 103/7.77 | 96/6.19 | 0.0122 | 0.0312 |
| 8 | Gal | Y=1474132593.196210X+6783328.803 | 0.9950 | 25~2000 | 1.55 | 5.01 | 3.34 | 2.39 | 3.28 | 3.48 | 98/5.10 | 102/4.18 | 0.0042 | 0.0140 |
| 9 | Ara | Y=112931320.654595X+276962.47680 | 0.9909 | 20~1000 | 2.62 | 5.32 | 3.34 | 6.19 | 2.75 | 3.81 | 96/3.05 | 98/1.34 | 0.0013 | 0.0042 |
| 10 | Xyl | Y=1735501217.681100X+91013.21785 | 0.9973 | 1.8~250.5 | 2.96 | 7.57 | 5.35 | 1.39 | 2.29 | 1.32 | 95/7.07 | 97/4.28 | 0.0019 | 0.0063 |
| 11 | Fuc | Y=570339596.956334X+8489353.6712 | 0.9945 | 0.1~124.6 | 2.57 | 3.79 | 6.85 | 2.29 | 0.74 | 1.09 | 96/9.55 | 99/6.18 | 0.0001 | 0.0002 |
| 12 | Sor | Y=105393541.322682X-14257.924620 | 0.9990 | 1.1~100.4 | 4.69 | 7.70 | / | / | / | / | 98/2.07 | 94/3.19 | 0.0002 | 0.0005 |
| Note: 1. In the column headers, “F” denotes flower and “B” denotes bulb. 2. DL-Arabinose (Ara), D- (+) -Xylose (Xyl), D-Ribose (Rib), L-Fucose (Fuc), L- (+) -Rhamnose (Rha), D- (+) -Sorbose (Sor), D-Fructose (Fru), D-Galactose (Gal), D-Mannose (Man), D- (+) -Glucose (Glc), D-Galacturonic acid (GalA), D-Glucuronic acid (GlcA). 3. Sample Information for Bulb (B-5) and Flower (F-4) of *Fritillaria unibracteata* var. *wabuensis*. 4. For sorbose, “/” indicates that no chromatographic peak was detected in the sample solutions used for the corresponding validation assessments. | | | | | | | | | | | | | | |

# **Table S6.** Current-batch applicability confirmation of linearity and instrumental precision for five alkaloids quantified using a previously validated UHPLC-QQQ-MS method

| NO. | Name | Regression equation | R^2^ | Linearity ranges (μg/L) | Precision RSD% |
| --- | --- | --- | --- | --- | --- |
| 1 | Sipeimine-3β-D-glucoside | Y=15090561.935221X+2010801.246504 | 0.9985 | 0.1~146.5 | 1.21 |
| 2 | Peimisine | Y=1295959960.333170X+826411.28233 | 0.9927 | 0.1~57.2 | 0.62 |
| 3 | Sipeimine | Y=1047134841.171450X+16323870.093 | 0.9945 | 0.01~617 | 1.69 |
| 4 | Peimine | Y=2709191992.433150X+2223164.59 | 0.9937 | 0.1~152.4 | 2.51 |
| 5 | Peiminine | Y=75910461.458095X+805466.569958 | 0.9949 | 0.1~741.2 | 0.68 |

# **Table S7.** Results of UHPLC-Q-TOF-MS analyses

| NO. | Name | Formula | RT (min) | ESI+/- | MS (*m/z*) | Product Ion (*m/z*) | Error  (ppm) | Structure class | Source | Level |
| --- | --- | --- | --- | --- | --- | --- | --- | --- | --- | --- |
| 1 | Arginine* | C_6_H_14_N_4_O_2_ | 1.21 | [M+H]^+^ | 175.1199 | 158.0900, 116.0648 | -5.40 | Amino acid | ALL | Level 1 |
| 2 | Anthranilic acid (Luo et al., 2022) | C_7_H_7_NO_2_ | 1.31 | [M+H]^+^ | 138.0555 | 94.0662, 93.0561, 92.0504 | -3.98 | Organic acid | ALL | Level 2 |
| 3 | Proline* | C_5_H_9_NO_2_ | 1.39 | [M+H]^+^ | 116.0699 | 70.0658 | 6.13 | Amino acid | ALL | Level 1 |
| 4 | L-Valine (Zhao et al., 2021) | C_5_H_11_NO_2_ | 1.62 | [M+H]^+^ | 118.0853 | 72.0818, 55.0550 | 8.16 | Amino acid | ALL | Level 2 |
| 5 | Adenine (Luo et al., 2022) | C_5_H_5_N_5_ | 1.69 | [M+H]^+^ | 136.0624 | 119.0363, 92.0253, 67.0301 | -4.56 | Nucleobase | ALL | Level 2 |
| 6 | Citric acid or isomer (Wojtanowski & Mroczek, 2018) | C_6_H_8_O_7_ | 1.69 | [M-H]^-^ | 191.0212 | 387.0098, 129.0200, 111.0102 | -7.68 | Organic acid | ALL | Level 2 |
| 7 | Nicotinic acid* | C_6_H_5_NO_2_ | 1.74 | [M+H]^+^ | 124.0400 | 78.0349, 53.0392 | -5.65 | Vitamin | ALL | Level 1 |
| 8 | Citric acid or isomer (Wojtanowski et al., 2018) | C_6_H_8_O_7_ | 1.75 | [M-H]^-^ | 191.0211 | 387.0090, 129.0205, 111.0115 | -7.47 | Organic acid | ALL | Level 2 |
| 9 | Uracil* | C_4_H_4_N_2_O_2_ | 2.17 | [M+H]^+^ | 113.0347 | 96.0082, 95.0235 | -1.3 | Nucleobase | ALL | Level 1 |
| 10 | Uridine (Struck-Lewicka et al., 2014) | C_9_H_12_N_2_O_6_ | 2.25 | [M+H]^+^ | 245.0781 | 113.0354, 96.0091, 70.0292 | -5.27 | Nucleoside | ALL | Level 2 |
| 11 | L-Tyrosine (Zhao et al., 2021) | C_9_H_11_NO_3_ | 2.40 | [M+H]^+^ | 182.0825 | 165.0551, 147.0447, 136.0766, 123.0451, 91.0551, 77.0392 | -7.35 | Amino acid | ALL | Level 2 |
| 12 | L-isoleucine (Zhao et al., 2021) | [C_6_H_13_NO_2_](https://pubchem.ncbi.nlm.nih.gov/#query=C6H13NO2) | 2.62 | [M+H]^+^ | 132.1029 | 86.0977, 69.0709, 57.0580 | -7.59 | Amino acid | ALL | Level 2 |
| 13 | L-leucine* | [C_6_H_13_NO_2_](https://pubchem.ncbi.nlm.nih.gov/#query=C6H13NO2) | 2.80 | [M+H]^+^ | 132.1028 | 86.0975, 69.0714, 57.0583 | -6.83 | Amino acid | ALL | Level 1 |
| 14 | Adenosine* | C_10_H_13_N_5_O_4_ | 2.87 | [M+H]^+^ | 268.1056 | 136.0624, 57.0343 | -5.88 | Nucleoside | ALL | Level 1 |
| 15 | Pyridoxine (Zhao et al., 2021) | C_8_H_11_NO_3_ | 2.94 | [M+H]^+^ | 170.0818 | 152.0716, 134.0612, 124.0770, 106.0666, 77.0397, 65.0387 | -3.73 | Vitamin | ALL | Level 2 |
| 16 | Fructosyl isoleucine (Dawood et al., 2023) | C_12_H_23_NO_7_ | 2.95 | [M+H]^+^ | 294.1549 | 276.1436, 258.1321 | -0.58 | Amino acid | ALL | Level 2 |
| 17 | Guanosine (Struck-Lewicka et al., 2014) | C_10_H_13_N_5_O_5_ | 3.06 | [M+H]^+^ | 284.1003 | 152.0572, 135.0310, 98.0608 | -4.79 | Nucleoside | ALL | Level 2 |
| 18 | 5'-[Deoxyadenosine](https://www.chemsrc.com/en/cas/4754-39-6_361275.html) (Luo et al., 2022) | C_10_H_13_N_5_O_3_ | 3.08 | [M+H]^+^ | 252.1104 | 136.0622, 117.0547 | -5.11 | Nucleoside | ALL | Level 2 |
| 19 | Phenylalanine (Luo et al., 2022) | C_9_H_11_NO_2_ | 4.10 | [M+H]^+^ | 166.0864 | 120.0810, 103.0542, 93.0707, 77.0396 | -0.88 | Amino acid | ALL | Level 2 |
| 20 | Fructosyl phenylalanine (Dawood et al., 2023) | C_15_H_21_NO_7_ | 4.37 | [M+H]^+^ | 328.1408 | 310.1273, 292.1180, 264.1226 | -5.26 | Amino acid | ALL | Level 2 |
| 21 | Protocatechuic acid* | C_7_H_6_O_4_ | 5.05 | [M-H]^-^ | 153.0203 | 109.0308, 108.0229, 65.0037, 53.0309 | -6.28 | Organic acid | ALL | Level 1 |
| 22 | 6-O-caffeoyl-α-D-fructofuranosyl-(2-1)-α-D-glucopyranoside (Chen et al., 2014) | C_21_H_28_O_14_ | 5.76 | [M-H]^-^ | 503.1435 | 485.1342, 341.1116, 323.0799, 281.0735, 221.0476, 179.0349, 161.0246 | -5.69 | Phenylpropanoids | A | Level 2 |
| 23 | 5'-Deoxy-5'-methylthio adenosine (Lu et al., 2017) | C_11_H_15_N_5_O_3_S | 6.58 | [M+H]^+^ | 298.0981 | 136.0626, 127.0211, 119.1064, 97.0295 | -4.25 | Nucleoside | ALL | Level 2 |
| 24 | Aesculetin* | C_9_H_6_O_4_ | 8.35 | [M+H]^+^ | 179.0345 | 151.0392, 123.0449, 105.0346, 77.0395 | -3.45 | Coumarins | AB | Level 1 |
| 25 | Caffeic acid* | C_9_H_8_O_4_ | 8.87 | [M-H]^-^ | 179.0348 | 135.0463, 107.0513 | 1.01 | Organic acid | ALL | Level 1 |
| 26 | Acretoside (Chen et al., 2014) | C_21_H_28_O_13_ | 9.59 | [M-H]^-^ | 487.1482 | 307.0806, 163.0405, 145.0303 | -5.09 | Sucrose ester | ALL | Level 2 |
| 27 | Sipeimine-3β-D-glucoside* | C_33_H_53_NO_8_ | 11.57 | [M+H]^+^ | 592.3876 | 574.3802, 412.3226, 138.1284,  112.1126 | -5.42 | Alkaloid | ALL | Level 1 |
| 28 | 1-O-glycerol ferulate (Ji, 2022) | C_13_H_16_O_6_ | 12.07 | [M-H]^-^ | 267.0870 | 252.0655, 175.0402, 149.0607, 133.0308 | 1.54 | Phenylpropanoids | ALL | Level 2 |
| 29 | Ferulic acid* | C_10_H_10_O_4_ | 12.18 | [M-H]^-^ | 193.0498 | 149.0623, 134.0373 | -1.38 | Organic acid | ALL | Level 1 |
| 30 | (2S)-3-acetoxy-2-(hexopyranosyloxy) propyl 3-(4-hydroxyphenyl) acrylate (Ji, 2022) | C_20_H_26_O_11_ | 12.24 | [M-H]^-^ | 441.1427 | 163.0408, 145.0308 | -5.57 | Phenylpropanoids | ALL | Level 2 |
| 31 | Sipeimine* | C_27_H_43_NO_3_ | 12.46 | [M+H]^+^ | 430.3343 | 412.3182, 138.1250 | -6.36 | Alkaloid | ALL | Level 1 |
| 32 | Edpetiline or isomer (Bora et al., 2023) | C_33_H_53_NO_8_ | 12.86 | [M+H]^+^ | 592.3874 | 574.3709, 287.0561 | -5.08 | Alkaloid | ALL | Level 2 |
| 33 | Peiminine* | C_27_H_43_NO_3_ | 13.99 | [M+H]^+^ | 430.3338 | 412.3234 | -5.19 | Alkaloid | ALL | Level 1 |
| 34 | Peimine* | C_27_H_45_NO_3_ | 14.28 | [M+H]^+^ | 432.3497 | 414.3390, 398.3053, 176.1433, 138.1117, 124.1120 | -5.75 | Alkaloid | ALL | Level 1 |
| 35 | Hyperoside (Zhao et al., 2021) | C_21_H_20_O_12_ | 14.57 | [M-H]^-^ | 463.0898 | 300.0289, 271.0265, 243.0298, 151.0046 | -3.45 | Flavonoids | ALL | Level 2 |
| 36 | Rutin and isomer (Zhao et al., 2021) | C_27_H_30_O_16_ | 14.71 | [M-H]^-^ | 609.1474 | 463.1465, 301.0357, 300.0289 | -2.12 | Flavonoids | A | Level 2 |
| 37 | Rutin and isomer (Zhao et al., 2021) | C_27_H_30_O_16_ | 14.93 | [M-H]^-^ | 609.1476 | 463.1469, 301.0352, 300.0295 | -2.44 | Flavonoids | AB | Level 2 |
| 38 | Edpetiline or isomer (Bora et al., 2023) | C_33_H_53_NO_8_ | 14.94 | [M+H]^+^ | 592.3874 | 574.3757 | -6.27 | Alkaloid | ALL | Level 2 |
| 39 | Yibeinoside A or isomer (Zhou et al., 2010) | C_33_H_53_NO_7_ | 15.13 | [M+H]^+^ | 576.3923 | 414.3340, 396.3289, 98.0981 | -4.90 | Alkaloid | ALL | Level 2 |
| 40 | 7-Hydroxycoumarin | C_9_H_6_O_3_ | 15.34 | [M+H]^+^ | 163.0386 | 135.0440, 105.0336 | 2.29 | Coumarins | ALL | Level 2 |
| 41 | Peimisine* | C_27_H_41_NO_3_ | 15.40 | [M+H]^+^ | 428.3182 | 410.3005, 393.2745, 337.2134 | -5.33 | Alkaloid | ALL | Level 1 |
| 42 | Yibeinoside A or isomer (Zhou et al., 2010) | C_33_H_53_NO_7_ | 15.58 | [M+H]^+^ | 576.3923 | 414.3359, 396.3254, 98.0967 | -4.90 | Alkaloid | ALL | Level 2 |
| 43 | 3′-O-Methylquercetin 3-galactoside (Zhao et al., 2021) | C_22_H_22_O_12_ | 15.75 | [M-H]^-^ | 477.1048 | 449.1082, 357.0629, 314.0444, 243.0318 | -1.99 | Flavonoids | A | Level 2 |
| 44 | Astragalin (Wojtanowski et al., 2018) | C_21_H_20_O_11_ | 15.76 | [M-H]^-^ | 447.0947 | 285.0398, 284.0355, 255.0280, 227.0376 | -3.16 | Flavonoids | AB | Level 2 |
| 45 | Litseagermacrane (Wojtanowski et al., 2018) | C_15_H_24_O_2_ | 15.82 | [M+H]^+^ | 237.1864 | 219.1752, 167.1069, 151.1125, 135.1183, 107.0865 | -6.32 | Terpenoids | A | Level 2 |
| 46 | Kaempferol (Zhao et al., 2021) | C_15_H_10_O_6_ | 16.04 | [M-H]^-^ | 285.0419 | 151.0042, 135.0438, 133.0302, 83.0147, 65.7258 | -5.03 | Flavonoids | AB | Level 2 |
| 47 | Isorhamnetin-3-glucoside (Zhao et al., 2021) | C_22_H_22_O_12_ | 16.29 | [M-H]^-^ | 477.1048 | 315.0499, 314.0423, 299.0218, 243.0286, 151.0031 | -1.99 | Flavonoids | AB | Level 2 |
| 48 | Narcissoside* | C_28_H_32_O_16_ | 16.44 | [M-H]^-^ | 623.1638 | 315.0523, 271.0265, 151.0046 | -5.04 | Flavonoids | AB | Level 1 |
| 49 | Isorhamnetin (Chen et al., 2021) | C_16_H_12_O_7_ | 16.45 | [M+H]^+^ | 317.0651 | 153.0174 | 1.52 | Flavonoids | ALL | Level 2 |
| 50 | Solasonine (Distl & Wink, 2009) | C_45_H_73_NO_16_ | 16.74 | [M+H]^+^ | 884.5006 | 706.4636 | -0.44 | Alkaloid | ALL | Level 2 |
| 51 | Ebeiedinone (Zhou et al., 2010) | C_27_H_43_NO_2_ | 16.79 | [M+H]^+^ | 414.3383 | 396.3279, 119.0855, 98.0964, 95.0853 | -3.98 | Alkaloid | ALL | Level 2 |
| 52 | Puqienine E (Bora et al., 2023) | C_28_H_43_NO_3_ | 16.82 | [M+H]^+^ | 442.3340 | 424.3193 | -5.50 | Alkaloid | ALL | Level 2 |
| 53 | Quercitrin (Wojtanowski et al., 2018) | C_21_H_20_O_11_ | 17.15 | [M-H]^-^ | 447.0947 | 314.0425, 300.0244, 271.0195, 243.0306 | -3.16 | Flavonoids | AB | Level 2 |
| 54 | Hupeheninoside (Zhou et al., 2010) | C_33_H_55_NO_7_ | 17.18 | [M+H]^+^ | 578.4079 | 398.3412, 98.0975, 85.0291, 69.0339 | -4.80 | Alkaloid | ALL | Level 2 |
| 55 | β-Solanine or isomer (Jiang et al., 2024) | C_39_H_63_NO_11_ | 17.62 | [M+H]^+^ | 722.4513 | 560.3944, 398.3442, 380.3320, 98.0976 | -5.42 | Alkaloid | ALL | Level 2 |
| 56 | Spirosol-5-en-3-ol 4-O-α-L-rhamnopyranosyl-(1-6)-β-D-glucopyranoside or isomer (Ji, 2022) | C_39_H_63_NO_11_ | 17.73 | [M+COOH]^-^ | 766.4400 | 720.4349, 179.0563, 161.0461 | -2.43 | Alkaloid | ALL | Level 2 |
| 57 | Quercetin* | C_15_H_10_O_7_ | 18.23 | [M+H]^+^ | 303.0518 | 274.0457, 257.0422, 229.0483, 201.0508, 153.0178 | -6.19 | Flavonoids | AB | Level 1 |
| 58 | β-Solanine or isomer (Jiang et al., 2024) | C_39_H_63_NO_11_ | 18.41 | [M+H]^+^ | 722.4513 | 560.3971, 398.3421, 380.3334, 98.0972 | -5.42 | Alkaloid | ALL | Level 2 |
| 59 | Formononetin-7-O-glucoside (Ji, 2022) | C_22_H_22_O_9_ | 19.01 | [M-H]^-^ | 429.1216 | 253.0737, 193.0510, 161.0249 | -5.80 | Flavonoids | ALL | Level 2 |
| 60 | Demissidine-3-O-β-D-glucopyranosyl (1-4) glucopyranoside or isomer | C_39_H_65_NO_11_ | 19.24 | [M+COOH]^-^ | 768.4576 | 722.4508, 161.0468, 101.0255 | -5.02 | Alkaloid | AB | Level 2 |
| 61 | Luteolin* | C_15_H_10_O_6_ | 19.29 | [M-H]^-^ | 285.0413 | 133.0299 | -6.81 | Flavonoids | AB | Level 1 |
| 62 | α-Solamarine or isomer (Jiang et al., 2024) | C_45_H_73_NO_16_ | 21.18 | [M+H]^+^ | 884.5045 | 738.4437, 414.3375 | -4.85 | Alkaloid | ALL | Level 2 |
| 63 | Puqiedinone (Zhou et al., 2010) | C_27_H_43_NO_2_ | 21.26 | [M+H]^+^ | 414.3383 | 396.3271, 119.0845, 105.0703, 95.0844, 81.0706 | -3.98 | Alkaloid | ALL | Level 2 |
| 64 | Podophyllotoxin (Ji, 2022) | C_22_H_22_O_8_ | 21.55 | [M-H]^-^ | 413.1269 | 219.0705, 193.0535, 175.0393, 134.0301 | -6.54 | Lignans | AB | Level 2 |
| 65 | Puqienine A (Zhou et al., 2010) | C_28_H_47_NO_3_ | 21.74 | [M+H]^+^ | 446.3648 | 428.3525, 253.1960, 145.1000, 107.0872, 105.0697, 98.0956, 95.0856, 81.0710 | -4.33 | Alkaloid | ALL | Level 2 |
| 66 | Imperialine N-oxide (Bora et al., 2023) | C_27_H_43_NO_4_ | 21.77 | [M+H]^+^ | 446.3290 | 428.3151 | -5.65 | Alkaloid | ALL | Level 2 |
| 67 | Ebeienine (Zhou et al., 2010) | C_27_H_43_NO_2_ | 22.26 | [M+H]^+^ | 414.3383 | 396.3273, 98.0855, 81.0711, 67.0538 | -3.98 | Alkaloid | ALL | Level 2 |
| 68 | Spirosol-5-en-3-ol 4-O-α-L-rhamnopyranosyl- (1-6)-β-D-glucopyranoside or isomer (Ji, 2022) | C_39_H_63_NO_11_ | 23.13 | [M+COOH]^-^ | 766.4400 | 720.4359, 179.0562, 161.0474 | -2.43 | Alkaloid | ALL | Level 2 |
| 69 | α-Solamarine or isomer (Jiang et al., 2024) | C_45_H_73_NO_16_ | 23.36 | [M+H]^+^ | 884.5045 | 738.4443, 414.3394 | -4.85 | Alkaloid | ALL | Level 2 |
| 70 | Demissidine-3-O-β-D-glucopyranosyl (1-4) glucopyranoside or isomer (Ji, 2022) | C_39_H_65_NO_11_ | 24.95 | [M+COOH]^-^ | 768.4576 | 722.4519, 161.0468, 101.0252 | -5.02 | Alkaloid | ALL | Level 2 |
| 71 | Pinellic acid (Ji, 2022) | C_18_H_34_O_5_ | 28.07 | [M-H]^-^ | 329.2354 | 229.1459, 211.1363, 171.1039 | -6.21 | Organic acid | ALL | Level 2 |
| 72 | 2β, 3β, 14-Trihydroxy-cholest-7-en-6-one 3-O-α-L-rhamnopyranosyl-(1-3)-[β-D-glucopyranosyl-(1-2)]-β-D-glucopyranoside (Ji, 2022) | C_51_H_84_O_23_ | 31.01 | [M+COOH]^-^ | 1109.5439 | 917.4755, 901.4921, 755.4196, | -5.03 | Saponins | A | Level 2 |
| 73 | (2α, 3β, 5α, 25R)-2-Hydroxyspirostan-3-O-α-L-rhamnopyranosyl-(1-2)-β-D-glucopyranosyl-(1-2)-β-D-glucopyranoside (Ji, 2022) | C_45_H_74_O_18_ | 31.89 | [M+COOH]^-^ | 947.4896 | 901.4845, 755.4216 | -4.30 | Saponins | A | Level 2 |
| 74 | α-Linolenic acid or isomer (Luo et al., 2022) | C_18_H_30_O_2_ | 37.26 | [M+H]^+^ | 279.2340 | 149.0243, 81.0707, 67.0549 | -7.70 | Organic acid | ALL | Level 2 |
| 75 | α-Linolenic acid or isomer (Luo et al., 2022) | C_18_H_30_O_2_ | 37.44 | [M+H]^+^ | 279.2340 | 149.0250, 81.0703, 67.0544 | -7.70 | Organic acid | ALL | Level 2 |
| 76 | 9-Hydroxy-10(E),12(Z)-octadecadienoic acid (Ji, 2022) | C_18_H_32_O_3_ | 38.67 | [M-H]^-^ | 295.2303 | 277.2204, 195.1410, 183.0128 | -8.21 | Organic acid | ALL | Level 2 |

Note: 1. “A” denotes compounds detected only in F-B; “AB” denotes compounds detected in both bulb samples (F-B and FW-B); “ALL” denotes compounds detected in all four groups (F-F, F-B, FW-F, and FW-B). 2. An asterisk (*) indicates a reference standard. Level 1 indicates annotation confirmed using an authentic reference standard; Level 2 indicates putative annotation based on accurate mass and literature-consistent MS/MS fragmentation evidence.

# **Fig. S8.** Fragmentation pattern of compound **29**

Organic acids are a class of compounds bearing acidic functional groups (e.g., carboxyl, sulfonic, sulfinic, or thio-carboxyl groups) and are introduced here to support MS/MS-based structural annotation. Ferulic acid is a high-value plant-derived compound (Li et al., 2025) and was used here to illustrate the MS/MS fragmentation behavior of organic acids.

In negative-ion mode, compound **29** showed a quasi-molecular ion at *m/z* 193.0498 [M-H]^-^, with major product ions at *m/z* 149.0623 and 134.0373. The fragment at *m/z* 149.0623 was generated by loss of CO_2_ from the precursor ion, followed by loss of a CH_3_ group to yield *m/z* 134.0373. Based on these fragmentation features, together with confirmation using an authentic standard and literature evidence (Wang et al., 2022), compound **29** was identified as ferulic acid (C_10_H_10_O_4_).

# **Fig. S9.** Fragmentation pattern of compound **40**

Coumarins are common plant metabolites with characteristic MS/MS fragmentation behavior (Yan et al., 2023). Here, 7-hydroxycoumarin was used to illustrate the MS/MS fragmentation behavior of coumarins.

In positive-ion mode, compound **40** exhibited a quasi-molecular ion at *m/z* 163.0386 [M+H]^+^, with major product ions at *m/z* 135.0440 and 105.0336. The fragment at *m/z* 135.0440 corresponded to loss of CO from the precursor ion, followed by radical loss to generate *m/z* 105.0336. Based on these fragmentation features, compound **40** was identified as 7-hydroxycoumarin (C_9_H_6_O_3_).

# **Fig. S10.** Fragmentation pattern of compound **17**

Nucleosides, as fundamental building blocks of genetic material, are ubiquitous endogenous metabolites and common constituents in plant-derived matrices. Here, guanosine was used to illustrate the MS/MS fragmentation behavior of nucleosides.

In positive-ion mode, compound **17** showed a quasi-molecular ion at *m/z* 284.1003 [M+H]^+^, with major product ions at *m/z* 152.0572 and 135.0310. The ion at *m/z* 152.0572 was generated by loss of C_5_H_8_O_4_ from the precursor ion, followed by loss of NH_3_ to yield *m/z* 135.0310. Based on these fragmentation features and literature evidence, compound **17** was identified as guanosine (C_10_H_13_N_5_O_5_).

# **Fig. S11.** Fragmentation pattern of compound **19**

Amino acids are organic compounds containing both basic amino and acidic carboxyl groups. Phenylalanine, an essential amino acid in humans, was selected in this study to illustrate the MS/MS fragmentation patterns of amino acids.

In positive-ion mode, compound **19** exhibited a quasi-molecular ion at *m/z* 166.0864 [M+H]^+^, with major product ions at *m/z* 120.0810 and 103.0542. The fragment at *m/z* 120.0810 was generated by loss of an HCOOH group from the precursor ion, followed by loss of NH_3_ to yield *m/z* 103.0542. Based on these fragmentation features and literature evidence (Wang et al., 2022), compound **19** was identified as phenylalanine (C_9_H_11_NO_2_).

# **Table S8.** Representative differential compounds between FW-B and FW-F

| NO. | Name | Formula | *t*_R_ (min) | ESI+ (*m/z*) | VIP | Sample group associated with the positive loadings |
| --- | --- | --- | --- | --- | --- | --- |
| 1 | Arginine | C_6_H_14_N_4_O_2_ | 1.21 | M+H 175.1199 | 2.75 | FW-B |
| 2 | 5'-Deoxy-5'-methylthio adenosine | C_11_H_15_N_5_O_3_S | 6.58 | M+H 298.0981 | 1.21 | FW-B |
| 3 | Sipeimine-3β-D-glucoside | C_33_H_53_NO_8_ | 11.57 | M+H 592.3876 | 1.71 | FW-B |
| 4 | Edpetiline or isomer | C_33_H_53_NO_8_ | 12.86 | M+H 592.3879 | 2.27 | FW-B |
| 5 | β-Solanine or its isomer | C_39_H_63_NO_11_ | 17.62 | M+H 722.4513 | 1.60 | FW-F |
| 6 | β-Solanine or its isomer | C_39_H_63_NO_11_ | 18.41 | M+H 722.4513 | 1.77 | FW-F |

# **Table S9.** Representative differential compounds between F-B and F-F

| NO. | Name | Formula | *t*_R_ (min) | ESI+ (*m/z*) | VIP | Sample group associated with the positive loadings |
| --- | --- | --- | --- | --- | --- | --- |
| 1 | Arginine | C_6_H_14_N_4_O_2_ | 1.21 | M+H 175.1199 | 1.67 | F-B |
| 2 | L-tyrosine | C_9_H_11_NO_3_ | 2.40 | M+H 182.0825 | 1.51 | F-B |
| 3 | L-isoleucine | C_6_H_13_NO_2_ | 2.62 | M+H 132.1029 | 1.45 | F-F |
| 4 | Hupeheninoside | C_33_H_55_NO_7_ | 17.18 | M+H 578.4079 | 1.83 | F-F |
| 5 | Fructosyl isoleucine | C_12_H_23_NO_7_ | 2.95 | M+H 294.1549 | 3.39 | F-F |
| 6 | Isorhamnetin | C_16_H_12_O_7_ | 16.45 | M+H 317.0651 | 2.48 | F-F |
| 7 | Fructosyl phenylalanine | C_15_H_21_NO_7_ | 4.37 | M+H 328.1408 | 3.39 | F-F |

# **Table S10.** Representative differential compounds between FW-B and F-B

| NO. | Name | Formula | *t*_R_ (min) | ESI+ (*m/z*) | VIP | Sample group associated with the positive loadings |
| --- | --- | --- | --- | --- | --- | --- |
| 1 | Edpetiline or isomer | C_33_H_53_NO_8_ | 12.86 | M+H 592.3874 | 4.15 | FW-B |
| 2 | Puqienine E | C_28_H_43_NO_3_ | 16.82 | M+H 442.3340 | 2.08 | F-B |
| 3 | Hupeheninoside | C_33_H_55_NO_7_ | 17.18 | M+H 578.4079 | 1.64 | F-B |
| 4 | β-Solanine or its isomer | C_39_H_63_NO_11_ | 18.41 | M+H 722.4513 | 3.85 | F-B |
| 5 | Solasonine | C_45_H_73_NO_16_ | 16.74 | M+H 884.5006 | 5.05 | F-B |

# **Table S11.** Representative differential compounds between FW-F and F-F

| NO. | Name | Formula | *t*_R_ (min) | ESI+ (*m/z*) | VIP | Sample group associated with the positive loadings |
| --- | --- | --- | --- | --- | --- | --- |
| 1 | Uracil | C_4_H_4_N_2_O_2_ | 2.17 | M+H 113.0347 | 1.07 | FW-F |
| 2 | L-isoleucine | C_6_H_13_NO_2_ | 2.62 | M+H 132.1029 | 2.12 | F-F |
| 3 | β-Solanine or its isomer | C_39_H_63_NO_11_ | 17.62 | M+H 722.4513 | 1.40 | FW-F |
| 4 | Fructosyl isoleucine | C_12_H_23_NO_7_ | 2.95 | M+H 294.1549 | 3.13 | F-F |

# **Fig. S12.** TIC chromatograms of B-5 (A) and STD (mixed reference standards) (B)

# **Fig. S13.** MRM chromatograms of 5 alkaloids

# **Table S12.** Quantitative results, group-level descriptive statistics, and statistical summary of five alkaloids in 36 batches using UHPLC-QQQ-MS (mg/g)

| Name | Sig | Sip | Pein | Peim | Pei | Name | Sig | Sip | Pein | Peim | Pei |
| --- | --- | --- | --- | --- | --- | --- | --- | --- | --- | --- | --- |
| F-1 | 0.0011 | 0.0006 | 0.0001 | 0.0001 | 0.0002 | B-3 | 0.0105 | 0.0243 | 0.0359 | 0.0036 | 0.0187 |
| F-2 | 0.0019 | 0.0007 | 0.0002 | 0.0001 | 0.0003 | B-4 | 0.0068 | 0.1060 | 0.3391 | 0.0112 | 0.0253 |
| F-3 | 0.0015 | 0.0015 | 0.0002 | 0.0001 | 0.0002 | B-5 | 0.0069 | 0.9561 | 0.2154 | 0.0091 | 0.0219 |
| F-4 | 0.0042 | 0.1772 | 0.0118 | 0.0010 | 0.0030 | B-6 | 0.0001 | 0.0004 | 0.0483 | 0.0003 | 0.0004 |
| F-5 | 0.0048 | 0.2350 | 0.0485 | 0.0004 | 0.0354 | B-7 | 0.0002 | 0.0006 | 0.0346 | 0.0004 | 0.0007 |
| F-6 | 0.0003 | 0.0072 | 0.0071 | 0.0086 | 0.0017 | B-8 | 0.0016 | 0.0218 | 0.0469 | 0.0156 | 0.0209 |
| F-7 | 0.0014 | 0.0020 | 0.0003 | 0.0001 | 0.0004 | SB-9 | 0.0148 | 0.0297 | 0.0540 | 0.0014 | 0.0047 |
| F-8 | 0.0337 | 0.2600 | 0.0540 | 0.0001 | 0.0076 | SB-10 | 0.0002 | 0.0096 | 0.0178 | 0.0002 | 0.0004 |
| F-9 | 0.0016 | 0.0016 | 0.0002 | 0.0001 | 0.0002 | SB-11 | 0.0126 | 0.0251 | 0.0909 | 0.0098 | 0.0059 |
| F-10 | 0.0268 | 0.1740 | 0.0116 | 0.0004 | 0.0196 | SB-12 | 0.0180 | 0.1278 | 0.1561 | 0.0002 | 0.0279 |
| F-11 | 0.0017 | 0.0820 | 0.0075 | 0.0011 | 0.0103 | B-13 | 0.0121 | 0.0424 | 0.1428 | 0.0051 | 0.0095 |
| SF-12 | 0.0056 | 0.1043 | 0.0979 | 0.0013 | 0.0173 | B-14 | 0.0107 | 0.0672 | 0.1700 | 0.0079 | 0.0166 |
| SF-13 | 0.0002 | 0.0015 | 0.0011 | 0.0002 | 0.0003 | B-15 | 0.0113 | 0.0621 | 0.1529 | 0.0061 | 0.0211 |
| SF-14 | 0.0038 | 0.1121 | 0.3191 | 0.0033 | 0.0184 | B-16 | 0.0109 | 0.0535 | 0.1663 | 0.0073 | 0.0192 |
| SF-15 | 0.0048 | 0.2945 | 0.1062 | 0.0205 | 0.0493 | B-17 | 0.0001 | 0.0041 | 0.0131 | 0.0002 | 0.0005 |
| F-16 | 0.0005 | 0.0001 | 0.0081 | 0.0003 | 0.0052 | B-18 | 0.0001 | 0.0050 | 0.0089 | 0.0003 | 0.0009 |
| B-1 | 0.0057 | 0.0611 | 0.1685 | 0.0083 | 0.0149 | B-19 | 0.0011 | 0.0027 | 0.0097 | 0.0010 | 0.0045 |
| B-2 | 0.0037 | 0.0066 | 0.2759 | 0.0046 | 0.0052 | B-20 | 0.0005 | 0.0012 | 0.0131 | 0.0006 | 0.0068 |
| **Group-level descriptive statistics of quantified alkaloids** | | | | | | | | | | | |
| Compound | | Group | | n | Mean ± SD (mg/g) | | | Median (IQR) (mg/g) | | | |
| Sig | | F-B | | 10 | 0.0018 ± 0.0033 | | | 0.0003 (0.0001–0.0015) | | | |
|  |  | F-F | | 7 | 0.0012 ± 0.0006 | | | 0.0014 (0.0008–0.0016) | | | |
|  |  | FW-B | | 10 | 0.0110 ± 0.0038 | | | 0.0111 (0.0078–0.0125) | | | |
|  |  | FW-F | | 9 | 0.0095 ± 0.0120 | | | 0.0048 (0.0038–0.0056) | | | |
| Sip | | F-B | | 10 | 0.0076 ± 0.0086 | | | 0.0046 (0.0016–0.0089) | | | |
|  |  | F-F | | 7 | 0.0011 ± 0.0007 | | | 0.0015 (0.0006–0.0016) | | | |
|  |  | FW-B | | 10 | 0.1531 ± 0.2839 | | | 0.0616 (0.0452–0.0963) | | | |
|  |  | FW-F | | 9 | 0.1607 ± 0.0929 | | | 0.1740 (0.1043–0.2350) | | | |
| Pein | | F-B | | 10 | 0.0504 ± 0.0807 | | | 0.0262 (0.0131–0.0441) | | | |
|  |  | F-F | | 7 | 0.0015 ± 0.0029 | | | 0.0002 (0.0002–0.0007) | | | |
|  |  | FW-B | | 10 | 0.1656 ± 0.0756 | | | 0.1612 (0.1453–0.1696) | | | |
|  |  | FW-F | | 9 | 0.0737 ± 0.0996 | | | 0.0485 (0.0116–0.0979) | | | |
| Peim | | F-B | | 10 | 0.0027 ± 0.0048 | | | 0.0005 (0.0003–0.0029) | | | |
|  |  | F-F | | 7 | 0.0001 ± 0.0001 | | | 0.0001 (0.0001–0.0002) | | | |
|  |  | FW-B | | 10 | 0.0066 ± 0.0035 | | | 0.0076 (0.0054–0.0089) | | | |
|  |  | FW-F | | 9 | 0.0041 ± 0.0067 | | | 0.0011 (0.0004–0.0033) | | | |
| Pei | | F-B | | 10 | 0.0059 ± 0.0077 | | | 0.0027 (0.0006–0.0064) | | | |
|  |  | F-F | | 7 | 0.0010 ± 0.0019 | | | 0.0003 (0.0002–0.0003) | | | |
|  |  | FW-B | | 10 | 0.0167 ± 0.0079 | | | 0.0179 (0.0109–0.0217) | | | |
|  |  | FW-F | | 9 | 0.0181 ± 0.0156 | | | 0.0173 (0.0076–0.0196) | | | |
| **Statistical summary of targeted alkaloid quantification** | | | | | | | | | | | |
| Analyte | Kruskal-Wallis p | | Effect size (ε^2^) | | Significant pairwise comparisons after BH correction | | | | | | |
| Sig | 6.93 × 10^-5^ | | 0.590 | | F-F vs FW-B (q = 0.0019); F-B vs FW-F (q = 0.0177); F-B vs FW-B (q = 0.0002) | | | | | | |
| Sip | 5.98 × 10^-6^ | | 0.749 | | F-F vs FW-F (q = 9.66 × 10^-5^); F-F vs FW-B (q = 0.0007); F-B vs FW-F (q = 0.0007); F-B vs FW-B (q = 0.0060) | | | | | | |
| Pein | 3.52 × 10^-5^ | | 0.634 | | F-F vs F-B (q = 0.0178); F-F vs FW-F (q = 0.0178); F-F vs FW-B (q = 9.05 × 10^-6^); F-B vs FW-B (q = 0.0178); FW-F vs FW-B (q = 0.0376) | | | | | | |
| Peim | 0.0005 | | 0.459 | | F-F vs F-B (q = 0.0383); F-F vs FW-F (q = 0.0145); F-F vs FW-B (q = 0.0002) | | | | | | |
| Pei | 0.0002 | | 0.511 | | F-F vs FW-F (q = 0.0013); F-F vs FW-B (q = 0.0005); F-B vs FW-B (q = 0.0424) | | | | | | |

Note: 1. Sipeimine-3β-D-glucoside (Sig), Peimisine (Pein), Peimine (Peim), Sipeimine (Sip), and Peiminine (Pei). 2. Values are expressed as mg/g starting material. Group-level mean ± SD and median (IQR) were calculated from independent batch-level replicates within each sample group, rather than from technical replicate injections. SD, standard deviation; IQR, interquartile range. Pairwise comparisons were performed using Dunn’s post hoc test after the Kruskal–Wallis test, and p values were adjusted using the Benjamini–Hochberg method. 3. Kruskal–Wallis p values indicate overall differences among the four sample groups. ε^2^ denotes epsilon-squared effect size for the Kruskal–Wallis test. Pairwise comparisons were performed using Dunn’s post hoc test, and p values were adjusted using the Benjamini–Hochberg method. Only pairwise comparisons with BH-adjusted q < 0.05 are listed. 4. Group assignments were based on Table S1 (F-F, F-B, FW-F, and FW-B).

**Table S13.** GPC-RI-MALS analysis results of 36 batches of samples

| Name | *M*_n_ (kDa) | *Mw* (kDa) | PDI | Name | *M*_n_ (kDa) | *Mw* (kDa) | PDI |
| --- | --- | --- | --- | --- | --- | --- | --- |
| F-1 | 80.7 | 380.6 | 4.72 | B-3 | 825.6 | 3260.9 | 3.95 |
| F-2 | 55.1 | 464.3 | 6.54 | B-4 | 98.0 | 823.7 | 5.34 |
| F-3 | 55.5 | 388.2 | 6.99 | B-5 | 128.8 | 552.3 | 4.29 |
| F-4 | 141.8 | 828 | 5.84 | B-6 | 541.7 | 2578.1 | 4.76 |
| F-5 | 65.2 | 426.4 | 6.54 | B-7 | 607.5 | 3035.7 | 5.00 |
| F-6 | 73.5 | 156.2 | 2.13 | B-8 | 458.9 | 2126.6 | 4.63 |
| F-7 | 59.2 | 495.4 | 6.00 | SB-9 | 91.5 | 196.5 | 2.15 |
| F-8 | 58.0 | 392.8 | 6.77 | SB-10 | 590.1 | 2918.2 | 4.95 |
| F-9 | 70.8 | 304.5 | 4.30 | SB-11 | 69.2 | 169.4 | 2.45 |
| F-10 | 130.2 | 857.2 | 6.58 | SB-12 | 61.9 | 153.3 | 2.48 |
| F-11 | 118.7 | 645.1 | 5.43 | B-13 | 45.6 | 141 | 3.09 |
| SF-12 | 120.1 | 691.3 | 5.76 | B-14 | 29.0 | 119.7 | 4.13 |
| SF-13 | 65.1 | 286.2 | 4.40 | B-15 | 199.4 | 540.2 | 2.71 |
| SF-14 | 96.2 | 563.1 | 5.85 | B-16 | 226.5 | 1125.6 | 4.97 |
| SF-15 | 92.5 | 591.3 | 6.39 | B-17 | 159.8 | 969.5 | 4.82 |
| F-16 | 125.6 | 443 | 3.53 | B-18 | 276.4 | 1613.6 | 4.75 |
| B-1 | 27.6 | 133.1 | 4.82 | B-19 | 209.4 | 1329.3 | 4.92 |
| B-2 | 165.1 | 1012.2 | 6.13 | B-20 | 153.1 | 903.0 | 4.59 |
| Note: Statistical summary for PDI: Kruskal–Wallis p = 0.0091; ε^2^ = 0.267. Dunn’s post hoc test with Benjamini–Hochberg correction identified a significant pairwise difference between FW-F and FW-B (q = 0.0047). | | | | | | | |

**Fig. S14.** Box plot of *M*_w_ across 36 batches of samples

**Table S14.** UHPLC-QQQ-MS analysis results and statistical summary of 12 monosaccharides (mg/g)

| Batch | Fru | Man | Rha | Rib | GlcA | Glc | GalA | Gal | Ara | Xyl | Fuc | Sor |
| --- | --- | --- | --- | --- | --- | --- | --- | --- | --- | --- | --- | --- |
| F-1 | 1.65 | 3.63 | 21.68 | 3.84 | 0.93 | 25.23 | 104.18 | 39.45 | 35.85 | 3.41 | 1.26 | / |
| F-2 | 2.92 | 5.60 | 32.93 | 1.64 | 1.34 | 46.23 | 137.71 | 67.96 | 30.92 | 4.89 | 0.94 | / |
| F-3 | 4.62 | 6.15 | 31.00 | 1.20 | 1.09 | 46.96 | 103.65 | 61.17 | 24.04 | 3.91 | 0.72 | / |
| F-4 | 1.81 | 20.36 | 28.47 | 2.30 | 1.28 | 48.90 | 40.65 | 66.48 | 13.39 | 5.66 | 1.63 | / |
| F-5 | 0.51 | 6.94 | 20.11 | 1.28 | 1.35 | 24.93 | 132.93 | 59.68 | 27.78 | 4.77 | 1.01 | / |
| F-6 | 0.19 | 1.85 | 11.01 | 0.66 | 0.06 | 13.75 | 10.43 | 22.73 | 6.48 | 0.32 | 1.91 | / |
| F-7 | 1.28 | 1.91 | 17.83 | 1.43 | 0.70 | 20.72 | 42.53 | 31.08 | 17.72 | 1.47 | 1.36 | / |
| F-8 | 0.70 | 4.95 | 20.21 | 0.93 | 0.85 | 16.72 | 85.08 | 33.24 | 20.66 | 4.24 | 3.45 | / |
| F-9 | 0.84 | 1.04 | 11.65 | 2.29 | 0.73 | 12.79 | 76.01 | 29.38 | 17.08 | 1.75 | 0.43 | / |
| F-10 | 1.46 | 4.87 | 21.22 | 3.86 | 0.89 | 27.78 | 187.78 | 51.25 | 35.94 | 7.60 | 2.30 | / |
| F-11 | 0.98 | 16.11 | 23.65 | 1.89 | 0.57 | 40.29 | 39.64 | 62.41 | 20.90 | 6.18 | 1.63 | / |
| SF-12 | 2.16 | 8.29 | 22.29 | 1.56 | 1.16 | 30.26 | 39.36 | 57.68 | 15.65 | 3.96 | 1.65 | / |
| SF-13 | 1.85 | 5.82 | 36.28 | 0.86 | 1.80 | 61.82 | 99.21 | 74.96 | 24.79 | 5.16 | 0.88 | / |
| SF-14 | 1.26 | 6.21 | 24.62 | 1.23 | 1.23 | 62.63 | 45.69 | 65.01 | 19.28 | 4.56 | 1.10 | / |
| SF-15 | 3.80 | 15.57 | 27.83 | 4.11 | 1.72 | 46.54 | 60.30 | 79.37 | 40.61 | 12.13 | 2.06 | / |
| F-16 | 2.25 | 6.62 | 30.65 | 1.10 | 1.32 | 45.29 | 90.65 | 50.64 | 20.36 | 5.13 | 2.34 | / |
| B-1 | 2.50 | 0.93 | 0.79 | 0.55 | 0.04 | 126.97 | 1.36 | 7.66 | 4.39 | 0.76 | 0.07 | / |
| B-2 | 1.65 | 0.85 | 0.65 | 1.04 | 0.05 | 143.38 | 3.34 | 5.48 | 4.14 | 0.40 | 0.05 | / |
| B-3 | 2.62 | 1.15 | 0.72 | 1.19 | 0.04 | 135.54 | 2.41 | 7.53 | 4.43 | 0.48 | 0.07 | / |
| B-4 | 1.83 | 0.85 | 2.27 | 1.94 | 0.08 | 98.66 | 3.67 | 16.42 | 9.82 | 1.20 | 0.16 | / |
| B-5 | 3.34 | 1.53 | 2.09 | 0.59 | 0.15 | 146.25 | 3.68 | 19.66 | 8.09 | 1.74 | 0.09 | / |
| B-6 | 3.97 | 1.97 | 0.95 | 0.67 | 0.04 | 135.36 | 1.44 | 7.71 | 5.80 | 0.63 | 0.09 | / |
| B-7 | 1.85 | 0.76 | 0.97 | 0.39 | 0.10 | 141.83 | 3.90 | 8.14 | 6.93 | 0.69 | 0.09 | / |
| B-8 | 0.49 | 0.49 | 0.82 | 0.29 | 0.04 | 110.37 | 1.31 | 6.66 | 6.14 | 0.65 | 0.10 | / |
| SB-9 | 0.85 | 1.22 | 0.93 | 1.23 | 0.06 | 119.13 | 2.15 | 7.68 | 5.36 | 1.11 | 0.11 | / |
| SB-10 | 0.69 | 0.81 | 1.94 | 1.45 | 0.06 | 123.76 | 4.32 | 12.40 | 8.84 | 0.97 | 0.10 | / |
| SB-11 | 0.25 | 0.28 | 0.30 | 0.43 | 0.01 | 93.14 | 0.37 | 3.80 | 2.38 | 0.46 | 0.03 | / |
| SB-12 | 7.29 | 2.73 | 2.12 | 3.06 | 0.07 | 138.31 | 1.56 | 11.85 | 7.20 | 1.61 | 0.12 | / |
| B-13 | 1.55 | 0.46 | 0.78 | 0.48 | 0.03 | 93.28 | 1.63 | 7.25 | 4.18 | 0.56 | 0.08 | / |
| B-14 | 0.93 | 0.60 | 1.18 | 0.68 | 0.07 | 86.44 | 1.89 | 10.54 | 4.43 | 0.56 | 0.11 | / |
| B-15 | 1.14 | 0.35 | 0.94 | 0.46 | 0.06 | 70.53 | 1.29 | 6.51 | 2.69 | 0.25 | 0.07 | / |
| B-16 | 1.75 | 1.10 | 1.28 | 1.45 | 0.15 | 112.75 | 1.64 | 12.32 | 7.59 | 0.79 | 0.16 | / |
| B-17 | 0.68 | 0.46 | 0.40 | 0.71 | 0.03 | 83.17 | 0.53 | 5.15 | 2.97 | 0.32 | 0.04 | / |
| B-18 | 2.77 | 0.78 | 0.94 | 2.27 | 0.13 | 108.01 | 2.49 | 10.33 | 7.68 | 0.52 | 0.11 | / |
| B-19 | 0.94 | 0.44 | 0.51 | 1.07 | 0.05 | 70.14 | 0.91 | 3.55 | 3.13 | 0.31 | 0.06 | / |
| B-20 | 1.01 | 0.79 | 1.30 | 1.68 | 0.14 | 95.58 | 3.39 | 8.42 | 6.19 | 0.73 | 0.11 | / |
| **Statistical summary of monosaccharide composition** | | | | | | | | | | | | |
| Analyte | | Kruskal-Wallis p | | | Effect size (ε^2^) | | Significant pairwise comparisons after BH correction | | | | | |
| Fru | | 0.5132 | | | 0.000 | | None | | | | | |
| Man | | 2.72 × 10^-5^ | | | 0.651 | | F-F vs F-B (q = 0.0093); F-F vs FW-B (q = 0.0156); F-B vs FW-F (q = 0.0003); FW-F vs FW-B (q = 0.0004) | | | | | |
| Rha | | 7.21 × 10^-6^ | | | 0.737 | | F-F vs F-B (q = 0.0003); F-F vs FW-B (q = 0.0010); F-B vs FW-F (q = 0.0005); FW-F vs FW-B (q = 0.0022) | | | | | |
| Rib | | 0.0826 | | | 0.115 | | None | | | | | |
| GlcA | | 3.52 × 10^-5^ | | | 0.634 | | F-F vs F-B (q = 0.0014); F-F vs FW-B (q = 0.0018); F-B vs FW-F (q = 0.0014); FW-F vs FW-B (q = 0.0024) | | | | | |
| Glc | | 9.49 × 10^-6^ | | | 0.719 | | F-F vs F-B (q = 0.0007); F-F vs FW-B (q = 0.0013); F-B vs FW-F (q = 0.0007); FW-F vs FW-B (q = 0.0007) | | | | | |
| GalA | | 7.23 × 10^-6^ | | | 0.737 | | F-F vs F-B (q = 0.0007); F-F vs FW-B (q = 0.0003); F-B vs FW-F (q = 0.0021); FW-F vs FW-B (q = 0.0007) | | | | | |
| Gal | | 7.87 × 10^-6^ | | | 0.731 | | F-F vs F-B (q = 0.0008); F-F vs FW-B (q = 0.0034); F-B vs FW-F (q = 0.0002); FW-F vs FW-B (q = 0.0009) | | | | | |
| Ara | | 2.67 × 10^-5^ | | | 0.652 | | F-F vs F-B (q = 0.0012); F-F vs FW-B (q = 0.0012); F-B vs FW-F (q = 0.0012); FW-F vs FW-B (q = 0.0012) | | | | | |
| Xyl | | 0.0001 | | | 0.562 | | F-F vs F-B (q = 0.0028); F-F vs FW-B (q = 0.0213); F-B vs FW-F (q = 0.0007); FW-F vs FW-B (q = 0.0065) | | | | | |
| Fuc | | 5.24 × 10^-6^ | | | 0.758 | | F-F vs F-B (q = 0.0027); F-F vs FW-B (q = 0.0116); F-B vs FW-F (q = 4.89 × 10^-5^); FW-F vs FW-B (q = 0.0003) | | | | | |
| Sor | | Not calculated | | | Not calculated | | Not calculated | | | | | |
| Note: 1. Fru, fructose; Man, mannose; Rha, rhamnose; Rib, ribose; GlcA, glucuronic acid; Glc, glucose; GalA, galacturonic acid; Gal, galactose; Ara, arabinose; Xyl, xylose; Fuc, fucose; Sor, sorbose. 2. “/” or “Not calculated” indicates that sorbose was not detected in any of the 36 batches. 3. Kruskal–Wallis p values indicate overall differences among the four sample groups. ε^2^ denotes epsilon-squared effect size for the Kruskal–Wallis test. Pairwise comparisons were performed using Dunn’s post hoc test, and p values were adjusted using the Benjamini–Hochberg method. Only pairwise comparisons with BH-adjusted q < 0.05 are listed. 4. Group assignments were based on Table S1 (F-F, F-B, FW-F, and FW-B). | | | | | | | | | | | | |

# **Fig. S15. Spearman correlation heatmap between targeted alkaloids, GPC-RI-MALS-derived parameters, and quantifiable polysaccharide-derived monosaccharides across 36 Fritillaria batches**

Spearman correlation heatmap between targeted alkaloids, GPC-RI-MALS-derived parameters, and quantifiable polysaccharide-derived monosaccharides across 36 Fritillaria batches. Rows represent five targeted alkaloids and GPC-RI-MALS-derived *Mw*/PDI values, and columns represent quantifiable polysaccharide-derived monosaccharides. Sorbose was excluded because it was not detected in any of the 36 batches. Values in cells indicate Spearman’s ρ. Asterisks indicate Benjamini–Hochberg-adjusted significance levels: *q < 0.05, **q < 0.01, ***q < 0.001.

# References

Bora, P. S., Agrawal, P., Kaushik, N. K., Puri, S., Sahal, D., & Sharma, U. (2023). Antiplasmodial activity of the bulbs of Fritillaria cirrhosa D.Don (Syn: *Fritillaria roylei* Hook.): UPLC-IM-Q-TOF-MS/MS-based biochemometric approach for the identification of marker compounds. *Journal of Ethnopharmacology, 310*, *Article* 116389. <https://doi.org/10.1016/j.jep.2023.116389>

Chen, Y. H., Qi, J., Hua, J., & Yu, B. Y. (2014). Structural characterization and identification of major constituents in radix scrophulariae by HPLC coupled with electrospray ionization quadrupole time-of-flight tandem mass spectrometry. *Chinese Journal of Natural Medicines, 12*(1), 47-54. <https://doi.org/10.1016/S1875-5364(14)60009-1>

Chen, Z., Zhong, B., Barrow, C. J., Dunshea, F. R., & Suleria, H. A. R. (2021). Identification of phenolic compounds in Australian grown dragon fruits by LC-ESI-QTOF-MS/MS and determination of their antioxidant potential. *Arabian Journal of Chemistry, 14*(6), *Article* 103151. https://doi.org/10.1016/j.arabjc.2021.103151

Dawood, A. S., Sedeek, M. S., Farag, M. A., & Abdelnaser, A. (2023). Terfezia boudieri and Terfezia claveryi inhibit the LPS/IFN-gamma-mediated inflammation in RAW 264.7 macrophages through an Nrf2-independent mechanism. *Scientific reports, 13*(1), *Article* 10106. <https://doi.org/10.1038/s41598-023-35612-8>

Distl, M., & Wink, M. (2009). Identification and Quantification of Steroidal Alkaloids from Wild Tuber-Bearing Solanum Species by HPLC and LC-ESI-MS. *Potato Research, 52*(1), 79-104. <https://doi.org/10.1007/s11540-008-9123-0>

Ji, S. (2022). Analysis of chemical constituents differences between *Fritillaria cirrhosa* D. Don and *Fritillaria thunbergii* and its metabolomics based on PLC-Q-TOF-MS/MS. Master’s thesis, *Qinghai Normal University,* CNKI. <https://link.cnki.net/doi/10.27778/d.cnki.gqhzy.2022.000764>

Jiang, Y., Li, X., Zhao, W. J., Liu, F. J., Yang, L. L., Li, P., & Li, H. J. (2024). Integration of untargeted and pseudotargeted metabolomics reveals specific markers for authentication and adulteration detection of Fritillariae Bulbus using tandem mass spectrometry and chemometrics. [*Journal of Pharmaceutical and Biomedical Analysis*](https://www.sciencedirect.com/journal/journal-of-pharmaceutical-and-biomedical-analysis)*, 242*, *Article* 116013. <https://doi.org/10.1016/j.jpba.2024.116013>

Li, M., Li, Q., Lee, J.-K., Kalia, V. C., & Gong, C. (2025). Metabolic engineering strategies for food additive ferulic acid production. *Food Bioscience, 65*, *Article* 106108. https://doi.org/10.1016/j.fbio.2025.106108

Lu, Z., Wang, Q., Wang, M., Fu, S., Zhang, Q., Zhang, Z., . . . Gao, X. (2017). Using UHPLC Q-Trap/MS as a complementary technique to in-depth mine UPLC Q-TOF/MS data for identifying modified nucleosides in urine. *Journal of Chromatography B, 1051*, 108-117. <https://doi.org/10.1016/j.jchromb.2017.03.002>

Luo, Y., Cui, M., Wang, H., & Chen, S. (2022). Analysis of Chemical Composition in Different Parts of *Fritillaria thunbergii* Using UHPLC-QTOF-MS/MS. *Chinese* *Journal of Modern Applied Pharmacy, 39*, 1984-1991. https://doi.org/10.13748/j.cnki.issn1007-7693.2022.15.011

Struck-Lewicka, W., Kaliszan, R., & Markuszewski, M. J. (2014). Analysis of urinary nucleosides as potential cancer markers determined using LC-MS technique. [*Journal of Pharmaceutical and Biomedical Analysis*](https://www.sciencedirect.com/journal/journal-of-pharmaceutical-and-biomedical-analysis)*, 101*, 50-57. <https://doi.org/10.1016/j.jpba.2014.04.022>

Wang, M., Tian, W., Zhen, Y., Wu, L., Wang, Y., & Niu, L. (2022). Analysis of Chemical Constituents in Yi Medicine Micromeria biflora (Buch.-Ham.exD.Don) Benth.by UPLC-Q-TOF/MS. *Chinese Journal of Pharmaceutical Sciences, 57*(20), 1717-1725. https://doi.org/[10.11669/cpj.2022.20.005](https://doi.org/10.11669/cpj.2022.20.005)

Wojtanowski, K. K., & Mroczek, T. (2018). Study of a complex secondary metabolites with potent anti-radical activity by two dimensional TLC/HPLC coupled to electrospray ionization time-of-flight mass spectrometry and bioautography. *Analytica Chimica Acta, 1029*, 104-115. <https://doi.org/10.1016/j.aca.2018.03.066>

Yan, J., Wang, H., Zhang, L., Cao, G., Fu, C., & Tian, Z. (2023). Component Analysis of Fraxini Cortex Based on UPLC-Q-Exactive Orbitrap MS. *Chinese* *Journal of Modern Applied Pharmacy. 40*(08), 1009-1020. <https://doi.org/10.13748/j.cnki.issn1007-7693.20222148>

Zhao, L., Zhao, X., Xu, Y., Liu, X., Zhang, J., & He, Z. (2021). Simultaneous determination of 49 amino acids, B vitamins, flavonoids, and phenolic acids in commonly consumed vegetables by ultra-performance liquid chromatography-tandem mass spectrometry. *Food Chemistry, 344*, *Article* 128712. <https://doi.org/10.1016/j.foodchem.2020.128712>

Zhou, J. L., Xin, G. Z., Shi, Z. Q., Ren, M. T., Qi, L. W., Li, H. J., & Li, P. (2010). Characterization and identification of steroidal alkaloids in *Fritillaria species* using liquid chromatography coupled with electrospray ionization quadrupole time-of-flight tandem mass spectrometry. *Journal of Chromatography A, 1217*(45), 7109-7122. <https://doi.org/10.1016/j.chroma.2010.09.019>

1. ^⁎^ Corresponding authors.
   ^1^ These authors contributed equally to this work.

   E-mail addresses: gengzhao@scidc.org.cn (Zhao Geng); gouyan@scidc.org.cn (Yan Gou); 13956985695@139.com (Yazhong Zhang).
